# Associations of dietary factors and early-life agricultural occupational background with body composition among older adults with type 2 diabetes in suburban Chengdu: A cross-sectional study

**DOI:** 10.1097/MD.0000000000049534

**Published:** 2026-07-03

**Authors:** Jinjie Song, Jinfeng Gao, Yangyang Yan, Li Zhong, Xue Wang, Ximin Mou, Xiaoping Yu

**Affiliations:** a College of Laboratory Medicine, Chengdu Medical College, Chengdu, China; b Department of Clinical Nutrition, West China Longquan Hospital, Sichuan University (The First People’s Hospital of Longquanyi District, Chengdu), Chengdu, China; c Department of Clinical Nutrition, Chengdu Jinxin Geriatric Hospital, Chengdu, China; d School of Preclinical Medicine and School of Nursing, Chengdu University, Chengdu, China.

**Keywords:** body composition, dietary factors, occupational background, older adults, type 2 diabetes mellitus

## Abstract

This study aimed to investigate the associations of early-life agricultural occupational background and dietary factors with body composition among older adults with type 2 diabetes mellitus (T2DM) in suburban Chengdu. A total of 448 inpatients with T2DM admitted between January and August 2025 were enrolled. Group differences were assessed using nonparametric tests. Multivariable logistic regression was used to identify factors associated with abnormal skeletal muscle index (SMI). Multivariable linear regression was used in the primary analysis to examine factors associated with phase angle (PhA), and sensitivity analyses were performed using binary logistic regression after dichotomizing PhA according to sex-specific cutoff values. Of the participants, 213 (47.54%) had an agricultural occupational background. SMI and PhA differed significantly by sex, body mass index, age, and occupational background (all *P* < .05). In the agricultural group, poultry intake was associated with lower odds of abnormal SMI (odds ratio [OR] = 0.957, 95% confidence interval [CI] = 0.923–0.993, *P* = .019), and older age (OR = 1.132, 95% CI = 1.032–1.241, *P* = .009) and higher visceral fat area (OR = 1.044, 95% CI = 1.021–1.067, *P* < .001) were associated with higher odds. In the nonagricultural group, beef and mutton intake was associated with lower odds of abnormal SMI (OR = 0.969, 95% CI = 0.948–0.991, *P* = .006). Age was negatively associated with PhA (β = −0.029, 95% CI = −0.045 to −0.013, *P* < .001), while SMI was positively associated with PhA (β = 0.387, 95% CI = 0.224–0.551, *P* < .001) in the agricultural group. In the nonagricultural group, age, duration of diabetes, and visceral fat area were negatively associated with PhA, and body mass index was positively associated with PhA (all *P* < .001). Sensitivity analyses generally supported the robustness of associations for age and adiposity-related indicators. Older adults with T2DM and an agricultural occupational background had less favorable body composition. Age was the most consistent factor associated with PhA, while muscle-related factors were more prominent in the agricultural group and adiposity-related factors in the nonagricultural group, suggesting that both occupational background and dietary characteristics should be considered when developing individualized nutritional interventions for this population.

## 1. Introduction

Type 2 diabetes mellitus (T2DM) is a major global public health challenge, with its prevalence escalating significantly with age. Over the past decades, the incidence of T2DM has risen across nearly all regions and age groups, with particularly rapid growth in low- and middle-income countries.^[1]^ Globally, the prevalence remains low (<1%) among individuals under 20 years old but surges to over 20% in those aged 65 and above, reaching a peak of 24.4% in the 75 to 79 age group. In China, however, the disease burden is concentrated in the 60 to 69 age range. Specifically, the 60 to 64 (40.68%) and 65 to 69 (34.52%) age groups account for over 3-quarters of all T2DM cases among the elderly population.^[2]^ As a chronic noncommunicable disease, T2DM not only imposes substantial medical and economic burdens on older adults but also elevates the risk of disability and mortality due to common comorbidities, including cardiovascular disease, kidney disease, cognitive impairment, and sarcopenic obesity.

Skeletal muscle index (SMI) is an important indicator of skeletal muscle mass, and phase angle (PhA) reflects cell membrane integrity and cellular function and is widely regarded as a sensitive marker of nutritional status and body composition.^[3]^ In T2DM patients, reduced skeletal muscle mass has been associated with increased all-cause mortality,^[4]^ highlighting the importance of maintaining muscle mass and body composition in this population. In addition, lower limb muscle loss and increased visceral fat area (VFA) have been linked to the progression of diabetic nephropathy.^[5]^ Dietary factors are also closely associated with body composition, and these relationships may vary across regions and between urban and rural populations.^[6]^ In suburban Chengdu, dietary habits are characterized by high oil and salt intake, a relatively high proportion of animal fat consumption, and insufficient intake of dairy products and soy foods.^[7]^

Occupational background, especially early-life occupational experience, may shape long-term lifestyle patterns, including habitual food choices, physical activity, and health behaviors, which in turn may influence body composition in later life. In suburban areas of Chengdu, individuals with agricultural and nonagricultural occupational backgrounds may differ in their long-term dietary structure and metabolic health profiles. However, evidence on this issue remains limited, particularly among older adults with T2DM. Therefore, the present study aimed to examine differences in dietary intake between older adults with or without early-life agricultural occupational backgrounds, and to explore the associations of dietary factors and occupational background with body composition. By clarifying these relationships, this study may provide a basis for more targeted nutritional management and region-specific intervention strategies for older patients with T2DM.

## 2. Subjects and methods

### 2.1. Subjects

A total of 448 older inpatients with T2DM were recruited from a tertiary Grade A hospital in suburban Chengdu between January and August 2025. Data on demographic characteristics, dietary intake, lifestyle factors, and body composition were collected. Face-to-face questionnaire interviews on dietary habits and lifestyle were conducted by trained investigators. The inclusion criteria were as follows: age ≥60 years; no long-term bedridden status or difficulty with ambulation; ability to eat independently by mouth; and no severe hepatic or renal dysfunction or mental disorders. The exclusion criteria were as follows: age <60 years; bedridden status or wheelchair dependence; severe hepatic or renal dysfunction; presence of a cardiac pacemaker or metal implants; and inability to complete the questionnaire. All participants provided written informed consent, and the study was approved by the Medical Ethics Committee of the First People’s Hospital of Longquanyi District, Chengdu (no. 2024023).

### 2.2. Methods

#### 2.2.1. Sample size calculation

During the design stage of the overall project, the sample size was initially estimated based on the reported prevalence of sarcopenia among patients with T2DM in the general population. According to the prevalence reported by Yang et al^[8]^ (*P* = 29%), the required sample size was calculated using the formula $\$n=\overset{2}{\underset{1-\alpha /2}{Z}}\times P\left(1-P\right)/d^{2}\$$ With a two-sided significance level of $α=0.05$ and an allowable error of 4%, the estimated sample size was 495 participants. This estimate was primarily used for the broader project design and for reference in the initial sampling framework.

Since the present study specifically focused on older adults with T2DM living in suburban Chengdu, whose underlying prevalence may differ from that of the general population, an additional calculation was performed using a more comparable prevalence estimate. Based on the prevalence of 21% reported by Wu et al,^[9]^ the minimum required sample size for the present study was recalculated as 399 participants using the same formula. Ultimately, 448 participants were included in the final analysis.

#### 2.2.2. Survey methods

**Figure 1.**
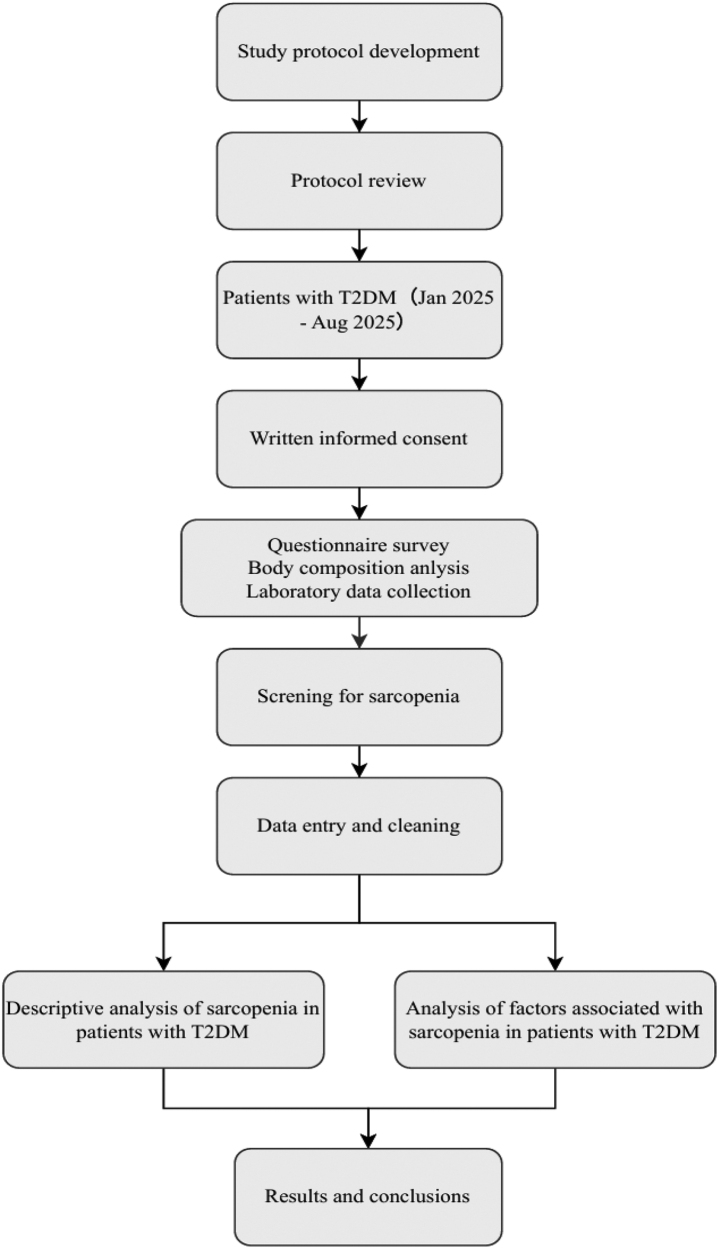
Study flowchart. T2DM = type 2 diabetes mellitus.

Participants were selected from T2DM inpatients at a tertiary Grade A hospital using convenience sampling. Paper-based questionnaires were completed by the participants with guidance from trained investigators. Biochemical parameters and body composition measurements were extracted from the hospital information system. Body composition was assessed using the InBody S10 body composition analyzer. The study flowchart is shown in Figure 1.

#### 2.2.3. Questionnaire design and definition of relevant indicators

Dietary intake over the previous year was assessed using a food frequency questionnaire adapted with reference to the relevant literature by Liu et al.^[10]^ Given the possibility of recall error among older hospitalized patients, several measures were taken to reduce recall bias. First, a simplified food frequency questionnaire with established reliability and validity was used to improve feasibility and comprehension. Second, all investigators received standardized training and assisted participants in completing the questionnaire by referring to usual dietary habits and, when necessary, family members’ recollections. Third, calibrated food models were used during the interviews to support portion size estimation.

Occupational information was collected retrospectively based on the participant’s primary occupation before retirement or, for those still working, their current main occupation. Retirement was defined as 60 years for men and 50 years for women according to the local policy context. Occupational information was primarily self-reported and was further verified with the assistance of family members when necessary. Because detailed information on job tasks, duration of employment, and exposure intensity was not consistently available for all participants, occupational classification was determined according to the type of occupation in which the participant had spent more than 10 years or more than 40% of his or her working life. If neither criterion was met, classification was based on the participant’s last main occupation before the survey.

Participants were then classified into 2 broad groups: an agricultural occupational background group and a nonagricultural occupational background group. The agricultural group included individuals mainly engaged in rice cultivation, vegetable cultivation, poultry breeding, and other agriculture-related work. The nonagricultural group included workers, professional and technical personnel, civil servants, and individuals engaged in other nonagricultural occupations.

### 2.3. Statistical analysis

Data management and statistical analyses were performed using SPSS version 26.0 (IBM Corporation) and R version 4.3.2 (R Foundation for Statistical Computing). Continuous variables are presented as mean ± standard deviation, and between-group comparisons were conducted using the Mann–Whitney *U* test. Categorical variables are expressed as counts (n) and percentages (%). Missing data accounted for <5% of the dataset and mainly involved certain physiological and biochemical indicators, primarily because some patients did not undergo the corresponding examinations. Given the low proportion of missing data, missing values were imputed using the random forest imputation method.

To examine the associations between dietary factors and body composition parameters, binary logistic regression was used for SMI, and multivariable linear regression was used in the primary analysis for PhA. Low SMI was defined according to the Asian Working Group for Sarcopenia 2019 criteria.^[11]^ PhA was analyzed as a continuous variable in the primary analysis because no universally accepted cutoff value has been established for the present study population. In the sensitivity analysis, PhA was also dichotomized using sex-specific cutoff values (<4.65° for men and <4.25° for women) derived from a Korean study conducted in an older Asian population.^[12]^ Because validated Chinese-specific reference cutoff values for PhA in older adults with T2DM are currently limited, we used these Asian population-based thresholds only for sensitivity analysis to examine the robustness of the main findings. Binary logistic regression was then applied to assess the robustness of main findings. Effect estimates were presented as β coefficients with 95% confidence intervals (CIs) for linear regression and odds ratios (OR) with 95% CI for logistic regression. All models were adjusted for potential confounders, including age, sex, body mass index (BMI), and other clinically relevant covariates. A two-tailed *P* < .05 was considered statistically significant.

## 3. Results

### 3.1. Baseline characteristics of the study participants

A total of 448 participants aged 60 years and older were included in the final analysis. Since the minimum required sample size for the present study was 399, the final analytical sample met the sample size requirement. Of the participants, 165 (36.83%) were male and 283 (63.17%) were female. In terms of occupational background, 213 participants (47.54%) had an agricultural occupational background, while 235 (52.46%) had a nonagricultural occupational background. Stratified by age, 222 participants (49.55%) were aged 60 to 69 years, 189 (42.19%) were aged 70 to 79 years, and 37 (8.26%) were aged 80 years and older (Table 1).^[13]^

**Table 1:** Demographic characteristics of older patients with T2DM in the agricultural and nonagricultural groups.

	Agricultural group			Nonagriculture group		
	N	Percentage within group (%)	Percentage of total sample (%)	N	Percentage within group (%)	Percentage of total sample (%)
Sex						
Male	42	19.72	9.38	123	52.34	27.45
Female	171	80.28	38.17	112	47.66	25.00
Age (yr)						
60–69	85	39.91	18.97	137	58.30	30.58
70–79	119	55.86	26.56	70	29.79	15.63
≥80	9	4.23	2.01	28	11.91	6.25
Household registration						
Urban	13	6.10	2.90	148	62.98	33.04
Nonurban	200	93.90	44.64	87	37.02	19.42
BMI (kg/m^2^)						
Low BMI	21	9.86	4.69	10	4.25	2.23
Normal BMI	132	61.97	29.46	155	65.96	34.60
High BMI	60	28.17	13.39	70	29.79	15.63
WHR						
Low risk	27	12.68	6.03	60	25.53	13.39
Moderate risk	40	18.78	8.93	68	28.94	15.18
High risk	146	68.54	32.59	107	45.53	23.88
Smoking status						
Yes	31	14.55	6.92	85	36.17	18.97
No	182	85.45	40.63	150	63.83	33.48
Alcohol consumption						
Beer	3	1.41	0.67	7	2.98	1.56
Wine & other colored alcoholic beverages	5	2.35	1.12	4	1.70	0.89
Chinese Baijiu	23	10.80	5.13	66	28.09	14.73
Total	213			235		

WHR, with the calculation formula: waist circumference (cm) divided by hip circumference (cm). BMI < 18.5: low BMI; 18.5 ≤ BMI < 24.0: normal BMI; BMI ≥ 24.0: high BMI.

BMI = body mass index, T2DM = type 2 diabetes mellitus, WHR = waist-hip ratio.

### 3.2. Differences in body composition according to participant characteristics

Statistically significant differences in PhA, SMI, and VFA were observed according to sex, occupational background, and BMI (all *P* < .05). PhA and SMI differed by sex, BMI, occupational background, and age, with higher values generally observed in males, participants with higher BMI, those in the nonagricultural group, and younger individuals. VFA was higher in females and in the agricultural group (Table 2). Fat mass in the upper extremities, lower extremities, and trunk was significantly higher in females than in males (all *P* < .05; Table 3). However, no significant differences in regional fat mass were observed between the agricultural and nonagricultural groups. Regional muscle mass in the upper extremities, lower extremities, and trunk was significantly lower in females than in males and was significantly lower in the agricultural group than in the nonagricultural group (all *P* < .05; Table 4). Across age groups, regional muscle mass decreased significantly with age (Table 4), but regional fat mass did not differ significantly overall among age groups (Table 3).

**Table 2:** Comparison of body composition in T2DM patients by characteristics.

	Category, n (%)	PhA	*Z*	*P*	SMI	*Z*	*P*	VFA	*Z*	*P*
		*M* (P_25_ ~ P_75_)			*M* (P_25_ ~ P_75_)			*M* (P_25_ ~ P_75_)		
Sex			−7.671	<.01		−12.999	<.01		−7.825	<.01
Male	165 (36.83)	5.200 (4.700, 5.700)			7.100 (6.500, 7.100)			86.200 (66.800, 107.300)		
Female	283 (63.17)	4.600 (4.200, 5.000)			5.700 (5.300, 6.300)			118.200 (91.400, 143.700)		
Occupation			−3.829	<.01		−5.297	<.01		−2.670	<.01
Agricultural	213 (47.54)	4.700 (4.300, 5.100)			5.900 (5.400, 6.500)			110.500 (86.500, 138.400)		
Nonagricultural	235 (52.46)	4.900 (4.400, 5.400)			6.500 (5.650, 7.200)			101.100 (73.950, 126.500)		
BMI (kg/m^2^)				<.01			<.01			<.01
Low BMI	31 (6.92)	4.200 (3.850, 4.650)	−3.512 (normal BMI)		5.000 (4.800, 5.550)	−5.114 (normal BMI)		53.400 (43.750, 66.650)	−6.564 (normal BMI)	
Normal BMI	287 (64.06)	4.700 (4.300, 5.200)	−4.078 (high BMI)		6.000 (5.450, 6.700)	−7.024 (high BMI)		98.200 (75.850, 118.250)	−10.496 (high BMI)	
High BMI	130 (29.02)	5.000 (4.700, 5.500)	−4.834 (low BMI)		6.750 (6.200, 7.400)	−6.998 (low BMI)		139.600 (114.700, 174.200)	−8.105 (low BMI)	
Age group (yr)				<.01			<.01			<.01
60–69	222 (49.55)	5.000 (4.700, 5.500)	−6.582 (70–79)		6.450 (5.700, 7.200)	−4.528 (70–79)		101.050 (71.400, 125.200)	−2.979 (70–79)	
70–79	189 (42.19)	4.600 (4.200, 5.100)	−4.216 (≥80)		5.900 (5.400, 6.700)	1.391 (≥80)	.164	110.400 (86.600, 138.100)	−0.052 (≥80)	.958
≥80	37 (8.26)	4.100 (3.800, 4.5000)	−6.933 (60–69)		5.400 (5.100, 6.800)	−3.505 (60–69)		103.900 (77.600, 156.100)	−1.493 (60–69)	.135
Total	448									

*M* (P25–P75) represents median (25th–75th percentiles), and *Z*-statistics and *P* values are derived from the Mann–Whitney *U* test.

BMI = body mass index, SMI = skeletal muscle index, T2DM = type 2 diabetes mellitus, VFA = visceral fat area.

**Table 3:** Comparison of body composition in T2DM patients by characteristics.

	Category, n (%)	Upper extremity fat mass	*Z*	*P*	Lower extremity fat mass	*Z*	*P*	Trunk fat mass	*Z*	*P*
		*M* (P_25_ ~ P_75_)			*M* (P_25_ ~ P_75_)			*M* (P_25_ ~ P_75_)		
Sex			−6.692	<.01		−5.349	<.01		−4.098	<.01
Male	165 (36.83)	2.400 (1.800, 3.000)			5.400 (4.400, 6.400)			9.600 (7.200, 11.500)		
Female	283 (63.17)	3.000 (2.400, 3.900)			6.200 (5.200, 7.400)			10.400 (8.600, 12.900)		
Occupation			−1.796	.073		−0.917	.359		−0.862	.389
Agricultural	213 (47.54)	3.000 (2.400, 3.700)			6.000 (5.000, 7.200)			10.300 (8.100, 12.500)		
Nonagricultural	235 (52.46)	2.800 (2.100, 3.400)			5.900 (4.800, 7.000)			10.224 (8.250, 12.250)		
BMI (kg/m^2^)				<.01			<.01			<.01
Low BMI	31 (6.92)	1.400 (1.000, 1.900)	−6.932 (normal BMI)		3.400 (2.800, 4.250)	−7.645 (normal BMI)		4.900 (3.150, 6.400)	−7.660 (normal BMI)	
Normal BMI	287 (64.06)	2.600 (2.100, 3.100)	−11.418 (high BMI)		5.600 (4.800, 6.300)	−11.689 (high BMI)		9.500 (8.050, 10.900)	−12.645 (high BMI)	
High BMI	130 (29.02)	4.000 (3.100, 5.000)	−8.285 (low BMI)		7.400 (6.400, 8.700)	−8.506 (low BMI)		13.100 (11.900, 15.400)	−8.464 (low BMI)	
Age group (yr)				.091			.279			.461
60–69	222 (49.55)	2.800 (2.100, 3.400)	−2.136 (70–79)	.033	5.800 (4.800, 7.000)	−1.545 (70–79)	.122	10.224 (8.100, 12.200)	−1.252 (70–79)	.211
70–79	189 (42.19)	2.993 (2.400, 3.700)	−0.114 (≥80)	.909	6.000 (5.000, 7.200)	−0.0847 (≥80)	.397	10.300 (8.500, 12.400)	−0.456 (≥80)	.648
≥80	37 (8.26)	2.800 (2.300, 4.400)	−1.017 (60–69)	.309	5.900 (4.600, 7.200)	−0.021 (60–69)	.983	9.800 (7.700, 14.200)	0.134 (60–69)	.893
Total	448									

BMI = body mass index, T2DM = type 2 diabetes mellitus.

**Table 4:** Comparison of body composition in T2DM patients by characteristics.

	Category, n (%)	Upper extremity muscle mass	*Z*	*P*	Lower extremity muscle mass	*Z*	*P*	Trunk muscle mass	*Z*	*P*
		*M* (P_25_ ~ P_75_)			*M* (P_25_ ~ P_75_)			*M* (P_25_ ~ P_75_)		
Sex			−14.028	<.01		−14.871	<.01		−14.415	<.01
Male	165 (36.83)	4.750 (4.100, 5.330)			13.560 (12.160, 14.960)			20.200 (18.200, 21.900)		
Female	283 (63.17)	3.350 (2.885, 3.735)			9.570 (8.725, 10.755)			15.800 (14.250, 17.000)		
Occupation			−6.024	<.01		−7.684	<.01		−6.588	<.01
Agricultural	213 (47.54)	3.460 (2.960, 4.020)			9.890 (8.830, 11.290)			16.100 (14.500, 17.900)		
Nonagricultural	235 (52.46)	4.100 (3.350, 4.890)			12.100 (9.875, 14.215)			18.200 (15.750, 20.650)		
BMI (kg/m^2^)				<.01			<.01			<.01
Low BMI	31 (6.92)	2.780 (2.365, 3.425)	−4.487 (normal BMI)		9.010 (8.055, 10.385)	−3.246 (normal BMI)		14.200 (12.750, 16.150)	−4.179 (normal BMI)	
Normal BMI	287 (64.06)	3.530 (3.025, 4.440)	−6.487 (high BMI)		10.390 (9.065, 12.975)	−3.467 (high BMI)		16.300 (14.750, 19.450)	−5.823 (high BMI)	
High BMI	130 (29.02)	4.190 (3.650, 4.810)	−6.740 (low BMI)		11.370 (10.140, 13.650)	−4.933 (low BMI)		18.350 (16.600, 20.300)	−6.442 (low BMI)	
Age group (yr)				<.01			<.01			<.01
60–69	222 (49.55)	3.985 (3.360, 4.820)	−5.119 (70–79)		11.465 (9.810, 13.790)	−5.527 (70–79)		17.850 (15.900, 20.300)	−5.380 (70–79)	
70–79	189 (42.19)	3.480 (2.970, 4.210)	−0.353 (≥80)	.724	10.030 (8.910, 12.000)	−0.477 (≥80)	.633	16.100 (14.500, 18.400)	−0.363 (≥80)	.718
≥80	37 (8.26)	3.480 (2.810, 4.370)	−2.880 (60–69)		9.660 (8.670, 12.530)	−3.102 (60–69)		16.200 (14.200, 19.000)	−3.036 (60–69)	
Total	448									

BMI = body mass index, T2DM = type 2 diabetes mellitus.

### 3.3. Multivariable logistic regression analysis of factors associated with abnormal SMI in different occupational groups

Multivariable logistic regression analyses were conducted separately in the agricultural and nonagricultural groups to identify factors associated with abnormal SMI. In the agricultural group, older age (OR = 1.132, 95% CI = 1.032–1.241, *P* = .009) and higher VFA (OR = 1.044, 95% CI = 1.021–1.067, *P* < .001) were associated with higher odds of abnormal SMI, whereas higher BMI (OR = 0.405, 95% CI = 0.278–0.589, *P* < .001) and daily poultry intake (OR = 0.957, 95% CI = 0.923–0.993, *P* = .019) were associated with lower odds of abnormal SMI. No significant associations were observed for waist circumference (WC), hip circumference (HC), PhA, daily vegetable intake, daily pork intake, or low-density lipoprotein (all *P* > .05). In the nonagricultural group, higher BMI (OR = 0.569, 95% CI = 0.448–0.723, *P* < .001), segmental PhA (OR = 0.049, 95% CI = 0.012–0.196, *P* < .001), and daily beef and mutton intake (OR = 0.969, 95% CI = 0.948–0.991, *P* = .006) were associated with lower odds of abnormal SMI. No significant associations were observed for age, WC, HC, daily rice intake, daily flour intake, or daily dry bean intake (all *P* > .05; Tables 5, 6, and S1–S4, Supplemental Digital Content 1).

**Table 5:** Multivariable logistic regression analysis of factors associated with abnormal SMI in the agricultural group.

Characteristic	N	Event N	OR	95% CI	*P*
Age	213	99	1.132	1.032–1.241	.009**
BMI	213	99	0.405	0.278–0.589	<.001***
Waist circumference	213	99	1.007	0.926–1.094	.875
Hip circumference	213	99	0.935	0.857–1.019	.123
VFA (cm^2^)	213	99	1.044	1.021–1.067	<.001***
PhA (°)	213	99	0.632	0.311–1.284	.205
Average daily vegetable intake	213	99	0.999	0.996–1.001	.365
Average daily pork intake	213	99	1.001	0.995–1.008	.733
Average daily poultry intake	213	99	0.957	0.923–0.993	.019*
Low-density lipoprotein	213	99	1.346	0.804–2.255	.259

Model fit: null deviance = 294; null df = 212; log-likelihood = −76.1; AIC = 174; BIC = 211; deviance = 152; residual df = 202; no. obs. = 213.

BMI = body mass index, CI = confidence interval, OR = odds ratio, PhA = phase angle, SMI = skeletal muscle index, VFA = visceral fat area.

* *P* < .05.

** *P* < .01.

*** *P* < .001.

**Table 6:** Multivariable logistic regression analysis of factors associated with abnormal SMI in the nonagricultural group.

Characteristic	N	Event N	OR	95% CI	*P*
Age	235	102	1.040	0.981–1.101	.188
BMI	235	102	0.569	0.448–0.723	<.001***
Waist circumference	235	102	1.057	0.984–1.136	.130
Hip circumference	235	102	1.019	0.947–1.097	.614
Extracellular water ratio	235	102	0.000	0.000–0.000	.002**
Segmental phase angle (°)	235	102	0.049	0.012–0.196	<.001***
Average daily rice intake	235	102	0.999	0.996–1.002	.516
Average daily flour intake	235	102	0.997	0.990–1.005	.515
Average daily dry bean intake	235	102	0.974	0.943–1.005	.096
Average daily beef and mutton intake	235	102	0.969	0.948–0.991	.006**

Model fit: null deviance = 322; null df = 234; log-likelihood = −99.9; AIC = 222; BIC = 260; deviance = 200; residual df = 224; no. obs. = 235.

BMI = body mass index, CI = confidence interval, OR = odds ratio, SMI = skeletal muscle index.

** *P* < .01.

*** *P* < .001.

### 3.4. Multivariable linear regression analysis of factors associated with PhA in different occupational groups

Multivariable linear regression analyses were conducted separately in the agriculture and nonagricultural groups to identify factors associated with PhA. In the agricultural group, age was negatively associated with PhA (β = −0.029, 95% CI = −0.045 to −0.013, *P* < .001), whereas SMI was positively associated with PhA (β = 0.387, 95% CI = 0.224–0.551, *P* < .001). No significant associations were observed for sex, BMI, WC, HC, daily pork intake, or daily poultry intake (all *P* > .05). In the nonagricultural group, age (β = −0.03, 95% CI = −0.04 to −0.02, *P* < .001), duration of diabetes (β = −0.02, 95% CI = −0.02 to −0.01, *P* < .001), and VFA (β = −0.01, 95% CI = −0.01 to −0.01, *P* < .001) were negatively associated with PhA, while BMI was positively associated with PhA (β = 0.13, 95% CI = 0.08–0.18, *P* < .001). Regarding dietary factors, daily rice, flour, and pork intakes were significantly associated with PhA (*P* < .001, *P* = .047, and *P* = .024, respectively), although the corresponding regression coefficients were small after rounding. Vitamin D level was also significantly associated with PhA (*P* = .047), whereas sex and SMI were not significantly associated with PhA in this group. The primary analyses suggest that the correlates of PhA differed by occupational background, with muscle-related factors appearing more prominent in the agricultural group and adiposity- and disease-related factors more prominent in the nonagricultural group (Tables 7, 8, and S5–S8, Supplemental Digital Content 5).

**Table 7:** Multivariable linear regression analysis of factors associated with PhA in the agricultural group.

Characteristic	N	β	95% CI	*P*
Sex				
Male	42	–	–	
Female	171	0.040	−0.259 to 0.339	.794
Age	213	−0.029	−0.045 to −0.013	<.001***
BMI	213	0.004	−0.040 to 0.048	.864
Waist circumference	213	0.003	−0.014 to 0.019	.756
Hip circumference	213	−0.004	−0.021 to 0.013	.664
SMI	213	0.387	0.224 to 0.551	<.001***
Average daily pork intake	213	0.000	−0.001 to 0.002	.529
Average daily poultry intake	213	0.000	−0.006 to 0.006	.935

Model fit: *R*^2^ = 0.337; adjusted *R*^2^ = 0.311; sigma = 0.594; statistic = 12.9; *P* value = <.001; df = 8; log-likelihood = −187; AIC = 393; BIC = 427; deviance = 72.0; residual df = 204; no. obs. = 213.

BMI = body mass index, CI = confidence interval, PhA = phase angle, SMI = skeletal muscle index.

*** *P* < .001.

**Table 8:** Multivariable linear regression analysis of factors associated with PhA in the nonagricultural group.

Characteristic	N	β	95% CI	*P*
Sex				
Male	123	–	–	
Female	112	0.07	−0.13 to 0.27	.492
Age	235	−0.03	−0.04 to −0.02	<.001***
BMI	235	0.13	0.08 to 0.18	<.001***
SMI	235	0.12	−0.02 to 0.27	.101
Duration of diabetes	235	−0.02	−0.02 to −0.01	<.001***
VFA	235	−0.01	−0.01 to −0.01	<.001***
Average daily rice intake	235	0.00	0.00 to 0.00	<.001***
Average daily flour intake	235	0.00	0.00 to 0.00	.047*
Average daily pork intake	235	0.00	0.00 to 0.00	.024*
Vitamin D level	235	0.00	−0.01 to 0.00	.047*

Model fit: *R*^2^ = 0.628; adjusted *R*^2^ = 0.609; sigma = 0.472; statistic = 34.2; *P* value = <.001; df = 11; log-likelihood = −151; AIC = 327; BIC = 372; deviance = 49.6; residual df = 223; no. obs. = 235.

BMI = body mass index, CI = confidence interval, PhA = phase angle, SMI = skeletal muscle index, VFA = visceral fat area.

* *P* < .05.

*** *P* < .001.

### 3.5. Sensitivity analysis of factors associated with PhA in different occupational groups

When PhA was dichotomized using sex-specific cutoff values and reanalyzed using binary logistic regression, the overall pattern was broadly consistent with the primary analyses. Older age remained consistently associated with abnormal PhA across models. Adiposity-related indicators, including BMI, fat mass, body fat percentage, and VFA, also showed relatively robust associations, although the specific indicator retained in the multivariable model varied across sensitivity analyses. In contrast, associations for individual dietary variables and vitamin D were less consistent, suggesting that these findings should be interpreted cautiously (Tables 9, 10, and S9–S12, Supplemental Digital Content 9).

**Table 9:** Sensitivity analysis of factors associated with PhA in the agricultural group.

Characteristic	N	Event N	OR	95% CI	*P*
Age	213	60	1.163	1.058–1.277	.002**
BMI	213	60	0.753	0.520–1.090	.133
WC	213	60	1.030	0.936–1.134	.541
HC	213	60	1.010	0.919–1.111	.831
SMI	213	60	0.595	0.159–2.227	.441
BFM	213	60	0.463	0.289–0.744	.001**
BF%	213	60	0.739	0.586–0.931	.010*
VFA	213	60	1.188	1.106–1.275	<.001***
Average daily vegetable intake	213	60	0.998	0.996–1.001	.306

Null deviance = 253; null df = 212; log-likelihood = −70.2; AIC = 160; BIC = 194; deviance = 140; residual df = 203; no. obs. = 213.

BF% = body fat percentage, BFM = body fat mass, BMI = body mass index, CI = confidence interval, OR = odds ratio, PhA = phase angle, SMI = skeletal muscle index, VFA = visceral fat area, WC = waist circumference.

* *P* < .05.

** *P* < .01.

*** *P* < .001.

**Table 10:** Sensitivity analysis of factors associated with PhA in the nonagricultural group.

Characteristic	N	Event N	OR	95% CI	*P*
Age	235	54	1.113	1.048–1.181	<.001***
BMI	235	54	0.400	0.266–0.602	<.001***
SMI	235	54	1.863	0.883–3.928	.102
Duration of diabetes	235	54	1.076	1.016–1.140	.012*
VFA	235	54	1.065	1.037–1.093	<.001***
Average daily rice intake	235	54	0.995	0.990–0.999	.016*
Hb	235	54	1.013	0.986–1.042	.341
Urea	235	54	0.893	0.715–1.116	.321
Creatinine	235	54	0.969	0.943–0.996	.022*

Null deviance = 253; null df = 234; log-likelihood = −73.9; AIC = 170; BIC = 208; deviance = 148; residual df = 224; no. obs. = 235.

BMI = body mass index, CI = confidence interval, Hb = hemoglobin, OR = odds ratio, PhA = phase angle, SMI = skeletal muscle index, VFA = visceral fat area.

* *P* < .05.

*** *P* < .001.

## 4. Discussion

In this cross-sectional study, we investigated demographic characteristics, dietary intake, lifestyle factors, and body composition among older adults with T2DM attending a tertiary hospital in suburban Chengdu. Females accounted for a larger proportion of the study population. This pattern may reflect longer life expectancy and possible sex differences in healthcare-seeking behavior among older adults, although sex-specific patterns in T2DM prevalence vary across populations and age groups.^[1,2,14,15]^ Females also had higher regional fat mass and lower regional muscle mass, which is consistent with well-established sex-related differences in body composition.^[16,17]^ These differences are likely shaped by a combination of hormonal factors, metabolic characteristics, lifestyle behaviors, and social influences.^[18,19]^

Clear differences in body composition were also observed according to occupational background. Participants with a nonagricultural occupational background had higher SMI and PhA than those with an agricultural occupational background, indicating heterogeneity in body composition characteristics across occupational groups. These differences may reflect long-term variation in socioeconomic conditions, living environment, healthcare access, dietary habits, occupational workload, and other health-related behaviors.^[20–22]^ These findings suggest that occupational background may be an important contextual factor in understanding the relationship between nutritional status and body composition in older adults with T2DM.

The multivariable analyses further demonstrated that factors associated with abnormal SMI differed between occupational groups. In the agricultural group, older age and higher VFA were associated with greater odds of abnormal SMI, and higher BMI and daily poultry intake were associated with lower odds. In the nonagricultural group, higher BMI, segmental PhA, and daily beef and mutton intake were associated with lower odds of abnormal SMI. These indicate that the determinants of muscle-related outcomes may vary by occupational background and likely reflect occupational differences in long-term physical activity patterns, energy expenditure, dietary structure, and metabolic status.

The inverse association between BMI and abnormal SMI observed in both the agricultural and nonagricultural groups deserves particular attention. Although BMI is a crude anthropometric indicator that does not distinguish fat mass from lean mass, in older adults with T2DM, a relatively higher BMI may still indicate a larger overall body frame and greater lean tissue reserve, particularly when compared with individuals at the lower end of the BMI distribution.^[11,23]^ In this context, BMI may function as an indirect marker of preserved skeletal muscle mass and therefore as a protective factor for normal SMI. Similar findings have been reported in previous studies showing that lower BMI is associated with a higher prevalence of low muscle mass or sarcopenia, whereas a relatively higher BMI is linked to a lower probability of meeting low-muscle-mass criteria.^[4,8,9]^ Mechanistically, the association between BMI and SMI may reflect the composite nature of body weight. Individuals with greater body size often retain higher absolute muscle mass because of larger constitutive lean mass, greater habitual mechanical loading, and more favorable long-term energy availability.^[11,24]^ At the same time, this apparent protective effect should not be overinterpreted, because a higher BMI may also coexist with excess adiposity, fat infiltration, impaired muscle quality, and sarcopenic obesity, especially in older adults with diabetes.^[11,25,26]^

From a dietary perspective, the present findings suggest that the factors associated with abnormal SMI differed between the agricultural group and the nonagricultural group, and that these differences likely reflect broader occupational and social contexts rather than isolated effects of specific food items alone. In the agricultural group, higher poultry intake was independently associated with lower odds of abnormal SMI, whereas in the nonagricultural group, higher beef and mutton intake showed a similar inverse association. By contrast, staple foods and plant-based items included in the models were not significantly associated with abnormal SMI. This pattern suggests that, in older adults with T2DM, skeletal muscle mass may be more closely related to the adequacy and quality of dietary protein than to staple carbohydrate intake alone.^[11,23,27–29]^ Because aging and T2DM are accompanied by anabolic resistance, insulin resistance, chronic low-grade inflammation, and impaired muscle homeostasis, this further increases the importance of high-quality protein for muscle maintenance.^[23,27,28,30]^ Therefore, these associations may suggest that, within each group, higher intake of poultry or beef and mutton serves as an indicator of better overall dietary protein adequacy and higher nutritional density, rather than reflecting independent protective effects of these foods themselves.^[27–29,31]^

Importantly, these dietary associations should be interpreted within the occupational and social environments of the 2 groups. Individuals in the agricultural group are more likely to experience long-term manual labor, irregular work intensity, seasonal workload fluctuation, and relatively constrained dietary choices, all of which may shape both energy expenditure and habitual food intake.^[7,21,22,32]^ In such settings, poultry intake may represent a more accessible and sustainable source of animal protein. By contrast, in the nonagricultural group, beef and mutton intake may better capture a dietary pattern characterized by higher protein density, greater dietary diversity, and better economic access to nutrient-rich foods.^[21,22,29]^ In addition, the metabolic implications of animal-source food intake may also depend on food preparation, since cooking methods can influence dietary exposure to compounds such as advanced glycation end products and may modify cardiometabolic responses.^[33]^

One noteworthy finding was that older age was associated with higher odds of abnormal SMI in the agricultural group, which is consistent with the expected age-related decline in skeletal muscle mass. This result supports previous evidence that aging remains a major risk factor for adverse muscle-related outcomes in older adults.^[11]^ In the same group, higher VFA was also associated with a greater likelihood of abnormal SMI, further suggesting that visceral adiposity may coexist with reduced muscle mass and an unfavorable metabolic profile. This pattern may be biologically plausible in older adults with T2DM. Excess adiposity may reflect a metabolically adverse state characterized by chronic low-grade inflammation and insulin resistance, partly through dysregulated adipokine and cytokine production. In addition, altered crosstalk between adipose tissue and skeletal muscle may adversely affect muscle metabolism and maintenance, which could partly explain the observed association with adverse muscle-related outcomes.^[30,34]^

With respect to PhA, the associated factors also differed by occupational background. In the agricultural group, age was negatively associated with PhA, whereas SMI was positively associated with it, suggesting that better muscle mass may be linked to improved cellular integrity and functional status in this population.^[3,35–37]^ In contrast, in the nonagricultural group, age, duration of T2DM, and VFA were negatively associated with PhA, and BMI was positively associated with PhA. The sensitivity analyses showed a broadly similar pattern, particularly for age and adiposity-related indicators, supporting the robustness of the main findings. By contrast, associations of individual dietary variables and vitamin D with PhA were less consistent across models and should therefore be interpreted with caution. The β coefficients for average daily rice, flour, and pork intake with respect to PhA were close to zero. This may be partly explained by the scale of the dietary variables in the regression models, as these intakes were entered in g/d. Consequently, the coefficients reflect the change in PhA associated with only a 1-g/d increase in intake and are therefore expected to be numerically small. Furthermore, body composition indicators such as BMI, VFA, and SMI may lie on the pathway between dietary intake and PhA, or may be associated with both; adjustment for these variables may therefore have attenuated the independent associations of individual food items.^[38]^ In addition, measurement error inherent in recall-based dietary assessment may have further biased the estimates toward the null.^[39]^ Given that dietary variables were not consistently retained across sensitivity analyses, these findings should be interpreted with caution.

In the present study, SMI was positively associated with PhA in the agricultural group, but this association was attenuated and no longer statistically significant in the nonagricultural group after multivariable adjustment. This pattern may be biologically relevant given that PhA is increasingly regarded as an integrative indicator of cellular membrane integrity, hydration status, and body cell mass rather than a simple anthropometric surrogate.^[3,35]^ Recent normative data also suggest that PhA declines with age,^[12]^ which is consistent with the inverse association between age and PhA observed in both occupational strata in our analysis. One possible explanation is that, within the agricultural group, SMI may more closely reflect aspects of muscle-related tissue status that are relevant to PhA. This interpretation may be compatible with the activity characteristics reported in agricultural settings, where work often involves substantial physical demands and muscular loading,^[40,41]^ as well as with evidence suggesting that muscle quantity, muscle quality, and muscle function are interrelated but not interchangeable constructs.^[11,23,36,37]^ Accordingly, in this subgroup, a higher SMI may be more closely aligned with the physiological attributes captured by PhA.

By contrast, in the nonagricultural group, the regression coefficient for SMI remained positive but was no longer statistically significant after adjustment for other covariates. This finding does not exclude a potential association between SMI and PhA, but it suggests that such an association may be weaker or more susceptible to confounding in this subgroup. In our model, BMI, VFA, and duration of diabetes were independently associated with PhA, raising the possibility that adiposity-related and disease-related factors may account for a larger proportion of the variation in PhA among nonagricultural participants. This interpretation is also supported by the sensitivity analyses, in which adiposity-related indicators remained repeatedly associated with abnormal PhA. This interpretation is broadly consistent with previous evidence linking visceral adiposity to metabolic inflammation, insulin resistance, and altered adipose–muscle crosstalk.^[5,23,25,26,30,34]^ In this context, PhA in the nonagricultural group may reflect a more complex combination of tissue composition, metabolic burden, and cellular status, which could partly reduce the independent contribution of SMI in the fully adjusted model.

Lifestyle and social-contextual differences may also have contributed to the between-group heterogeneity observed in the present study, although these factors were not directly measured in full detail and therefore should be interpreted cautiously. Compared with the agricultural group, nonagricultural populations may show greater heterogeneity in sedentary exposure, occupational activity patterns, socioeconomic position, and dietary behavior.^[21,22,32]^ In addition, the inclusion of diet-related variables and vitamin D in the nonagricultural model suggests that nutritional factors may be relevant to PhA in this subgroup. This possibility is supported by prior evidence showing that adequate protein intake and vitamin D status are important for maintaining muscle mass and function.^[27–29,31,42]^ Overall, our findings suggest that the correlates of PhA may differ by occupational grouping; however, the underlying mechanisms remain to be clarified in future studies with more detailed assessment of physical activity, dietary intake, and metabolic status.

This study has several limitations. First, its cross-sectional design precludes any inference of causality between dietary factors and body composition. Second, dietary intake was self-reported and may therefore be subject to recall bias. Third, occupational background was classified into only 2 broad categories, which may not fully capture heterogeneity in job type, work intensity, employment duration, or occupational exposures. Occupational information was collected retrospectively and relied primarily on self-report, although family members assisted with verification when necessary. As a result, some degree of misclassification cannot be excluded. Fourth, although the proportion of missing data was low, a random forest imputation method was used, which may have reduced variability and introduced bias. Fifth, the study was conducted at a single tertiary hospital in suburban Chengdu using convenience sampling, which may limit the representativeness and generalizability of the findings. Sixth, we did not incorporate several potentially important confounding factors, such as medication use, physical activity levels, and detailed socioeconomic indicators, which could influence both dietary habits and body composition. Finally, we explored multiple dietary variables in our statistical models. Given the exploratory nature of this study, we did not apply formal multiple-testing corrections to the *P* values. Consequently, the potential risk of Type I errors cannot be completely ruled out. Therefore, the nominally statistically significant associations identified in our study should be interpreted with caution and viewed as hypothesis-generating, which require further validation in future large-scale prospective studies.

Future studies should address these limitations through prospective cohort designs and randomized nutritional intervention trials to clarify causal relationships between dietary factors and body composition. More detailed occupational data, including job content, duration, workload, and exposure characteristics, should also be collected using standardized methods. Furthermore, it is crucial for subsequent research to comprehensively evaluate and adjust for key covariates, including objective physical activity levels, medication history, and socioeconomic status. In addition, multicenter studies with more representative sampling strategies are needed to improve external validity. To reduce recall bias in dietary assessment, future research may also consider more rigorous dietary survey methods, such as repeated 24-hour dietary recalls over consecutive days.

## Author contributions

**Data curation:** Jinjie Song.

**Formal analysis:** Jinjie Song.

**Investigation:** Jinjie Song, Yangyang Yan, Xue Wang, Ximin Mou.

**Methodology:** Jinjie Song, Jinfeng Gao.

**Resources:** Jinjie Song, Jinfeng Gao.

**Writing – original draft:** Jinjie Song.

**Writing – review & editing:** Jinjie Song, Jinfeng Gao, Xiaoping Yu.

**Conceptualization:** Jinfeng Gao, Xiaoping Yu.

**Funding acquisition:** Jinfeng Gao, Li Zhong.

**Supervision:** Jinfeng Gao.

**Validation:** Jinfeng Gao.
























