# Supplementary material for: Associations of dietary factors and early-life agricultural occupational background with body composition among older adults with type 2 diabetes in suburban Chengdu: A cross-sectional study
Source: Medicine (Baltimore). 2026 Jul 3;105(27):e49534. doi: 10.1097/MD.0000000000049534 (PMC13337032; doi:10.1097/MD.0000000000049534)
Supplement: Supplementary file 1 [file medi-105-e49534-s001.docx]

**Supplementary Table 1.** Univariate and multivariate analysis of influencing factors (SMI Logistic regression) in the agricultural group.

|  | **Univariable** | | | | | | **Multivariable** | | | | | |
| --- | --- | --- | --- | --- | --- | --- | --- | --- | --- | --- | --- | --- |
|  | **N** | **Event N** | **Estimate** | **SE** | **95% CI** | ***P*** | **N** | **Event N** | **Estimate** | **SE** | **95% CI** | ***P*** |
| **Sex** |  |  |  |  |  |  |  |  |  |  |  |  |
| Male | 42 | 20 | — | — | — |  |  |  |  |  |  |  |
| Female | 171 | 79 | -0.0570305447 | 0.3449379716 | -0.7330965459, 0.6190354565 | 0.869 |  |  |  |  |  |  |
| **Age** | 213 | 99 | 0.0914045472 | 0.0272044525 | 0.0380848000, 0.1447242944 | <0.001*** | 213 | 99 | 0.1237799335 | 0.0471337766 | 0.0313994290, 0.2161604380 | 0.009** |
| **BMI** | 213 | 99 | -0.4544066131 | 0.0678754192 | -0.5874399901, -0.3213732361 | <0.001*** | 213 | 99 | -0.9042359035 | 0.1916935695 | -1.2799483958, -0.5285234112 | <0.001*** |
| **Systolic blood pressure** | 213 | 99 | -0.0027473460 | 0.0068013011 | -0.0160776511, 0.0105829591 | 0.686 |  |  |  |  |  |  |
| **Diastolic blood pressure** | 213 | 99 | -0.0200062522 | 0.0120953042 | -0.0437126128, 0.0037001085 | 0.098 |  |  |  |  |  |  |
| **WC** | 213 | 99 | -0.1238656396 | 0.0206505940 | -0.1643400600, -0.0833912192 | <0.001*** | 213 | 99 | 0.0066488123 | 0.0424155206 | -0.0764840805, 0.0897817051 | 0.875 |
| **HC** | 213 | 99 | -0.1711755543 | 0.0282979661 | -0.2266385486, -0.1157125599 | <0.001*** | 213 | 99 | -0.0676966319 | 0.0439403680 | -0.1538181707, 0.0184249069 | 0.123 |
| **duration of diabetes** | 213 | 99 | -0.0034101435 | 0.0190550877 | -0.0407574292, 0.0339371421 | 0.858 |  |  |  |  |  |  |
| **VFA** | 213 | 99 | -0.0144537591 | 0.0036609477 | -0.0216290847, -0.0072784334 | <0.001*** | 213 | 99 | 0.0427043804 | 0.0113953918 | 0.0203698229, 0.0650389379 | <0.001*** |
| **Extracellular water ratio** | 213 | 99 | -0.8026051392 | 1.3065480926 | -3.3633923447, 1.7581820663 | 0.539 |  |  |  |  |  |  |
| **PhA** | 213 | 99 | -1.8535333244 | 0.3130702692 | -2.4671397766, -1.2399268721 | <0.001*** | 213 | 99 | -0.4583737429 | 0.3613850402 | -1.1666754062, 0.2499279204 | 0.205 |
| **Average daily intake of rice** | 213 | 99 | -0.0012227087 | 0.0009033559 | -0.0029932536, 0.0005478363 | 0.176 |  |  |  |  |  |  |
| **Average daily intake of flour** | 213 | 99 | -0.0047251561 | 0.0028054794 | -0.0102237947, 0.0007734826 | 0.092 |  |  |  |  |  |  |
| **Average daily intake of other cereals** | 213 | 99 | 0.0009276280 | 0.0034911087 | -0.0059148193, 0.0077700753 | 0.790 |  |  |  |  |  |  |
| **Average daily intake of tubers** | 213 | 99 | -0.0043909817 | 0.0045828813 | -0.0133732639, 0.0045913005 | 0.338 |  |  |  |  |  |  |
| **Average daily intake of dairy products** | 213 | 99 | 0.0000340068 | 0.0011430450 | -0.0022063203, 0.0022743339 | 0.976 |  |  |  |  |  |  |
| **Average daily intake of eggs** | 213 | 99 | -0.0085672706 | 0.2563259764 | -0.5109569526, 0.4938224115 | 0.973 |  |  |  |  |  |  |
| **Average daily intake of dried beans** | 213 | 99 | 0.0162533260 | 0.0129568896 | -0.0091417110, 0.0416483630 | 0.210 |  |  |  |  |  |  |
| **Average daily intake of soy products** | 213 | 99 | -0.0000958401 | 0.0075611762 | -0.0149154731, 0.0147237928 | 0.990 |  |  |  |  |  |  |
| **Average daily intake of vegetables** | 213 | 99 | -0.0020729477 | 0.0008896800 | -0.0038166883, -0.0003292070 | 0.020* | 213 | 99 | -0.0012173263 | 0.0013426822 | -0.0038489351, 0.0014142824 | 0.365 |
| **Average daily intake of fruits** | 213 | 99 | -0.0000031910 | 0.0017776047 | -0.0034872323, 0.0034808503 | 0.999 |  |  |  |  |  |  |
| **Average daily intake of pork** | 213 | 99 | -0.0041041842 | 0.0019992631 | -0.0080226680, -0.0001857005 | 0.040* | 213 | 99 | 0.0011423977 | 0.0033549430 | -0.0054331697, 0.0077179651 | 0.733 |
| **Average daily intake of poultry** | 213 | 99 | -0.0384936988 | 0.0138377553 | -0.0656152007, -0.0113721968 | 0.005** | 213 | 99 | -0.0438362078 | 0.0187114732 | -0.0805100213, -0.0071623943 | 0.019* |
| **Average daily intake of beef and mutton** | 213 | 99 | -0.0017548104 | 0.0093430665 | -0.0200668843, 0.0165572634 | 0.851 |  |  |  |  |  |  |
| **Average daily intake of aquatic products** | 213 | 99 | -0.0264948459 | 0.0136696351 | -0.0532868384, 0.0002971466 | 0.053 |  |  |  |  |  |  |
| **Hemoglobin** | 213 | 99 | -0.0046439660 | 0.0071478656 | -0.0186535252, 0.0093655931 | 0.516 |  |  |  |  |  |  |
| **Albumin** | 213 | 99 | -0.0161389631 | 0.0276546431 | -0.0703410676, 0.0380631415 | 0.559 |  |  |  |  |  |  |
| **Prealbumin** | 213 | 99 | 0.0000784956 | 0.0024611036 | -0.0047451789, 0.0049021702 | 0.975 |  |  |  |  |  |  |
| **Urea** | 213 | 99 | -0.0078160676 | 0.0560617068 | -0.1176949938, 0.1020628586 | 0.889 |  |  |  |  |  |  |
| **Creatinine** | 213 | 99 | -0.0012875810 | 0.0055247194 | -0.0121158319, 0.0095406700 | 0.816 |  |  |  |  |  |  |
| **Vitamin D level** | 213 | 99 | -0.0004712924 | 0.0052801111 | -0.0108201200, 0.0098775351 | 0.929 |  |  |  |  |  |  |
| **Total cholesterol** | 213 | 99 | 0.2240143775 | 0.1158108900 | -0.0029707959, 0.4509995509 | 0.053 |  |  |  |  |  |  |
| **Triglycerides** | 213 | 99 | -0.0104541254 | 0.0503981002 | -0.1092325867, 0.0883243359 | 0.836 |  |  |  |  |  |  |
| **High-density lipoprotein** | 213 | 99 | -0.0484764856 | 0.3132901749 | -0.6625139452, 0.5655609740 | 0.877 |  |  |  |  |  |  |
| **Low-density lipoprotein** | 213 | 99 | 0.3619204008 | 0.1708396671 | 0.0270808060, 0.6967599955 | 0.034* | 213 | 99 | 0.2973410845 | 0.2631642823 | -0.2184514309, 0.8131335999 | 0.259 |
| **Alanine aminotransferase** | 213 | 99 | 0.0115303909 | 0.0075153853 | -0.0031994936, 0.0262602755 | 0.125 |  |  |  |  |  |  |
| **Aspartate aminotransferase** | 213 | 99 | 0.0138714972 | 0.0135687272 | -0.0127227194, 0.0404657138 | 0.307 |  |  |  |  |  |  |
| **HbA1c** | 213 | 99 | 2.1692421752 | 2.6331724486 | -2.9916809891, 7.3301653395 | 0.410 |  |  |  |  |  |  |
| **Fasting blood glucose** | 213 | 99 | 0.0282082629 | 0.0302300981 | -0.0310416405, 0.0874581664 | 0.351 |  |  |  |  |  |  |

1*p<0.05; **p<0.01; ***p<0.001

Abbreviations: CI = Confidence Interval, OR = Odds Ratio, SE = Standard Error, NA
