# Supplementary material for: Associations of dietary factors and early-life agricultural occupational background with body composition among older adults with type 2 diabetes in suburban Chengdu: A cross-sectional study
Source: Medicine (Baltimore). 2026 Jul 3;105(27):e49534. doi: 10.1097/MD.0000000000049534 (PMC13337032; doi:10.1097/MD.0000000000049534)
Supplement: Supplementary file 2 [file medi-105-e49534-s002.docx]

Supplementary Table 2. Variance Inflation Factor and Tolerance (PhA Logistic regression) in the agricultural group.

|  | VIF | VIF CI low | VIF CI high | SE factor | Tolerance | Tolerance CI low | Tolerance CI high |
| --- | --- | --- | --- | --- | --- | --- | --- |
| **Age** | 1.304351 | 1.156490 | 1.591918 | 1.142082 | 0.7666649 | 0.6281731 | 0.8646851 |
| **BMI** | 5.879963 | 4.697494 | 7.440588 | 2.424864 | 0.1700691 | 0.1343980 | 0.2128794 |
| **WC** | 2.578286 | 2.133046 | 3.198488 | 1.605704 | 0.3878545 | 0.3126477 | 0.4688131 |
| **HC** | 1.861749 | 1.578960 | 2.282664 | 1.364459 | 0.5371292 | 0.4380846 | 0.6333281 |
| **VFA** | 4.620531 | 3.718738 | 5.821445 | 2.149542 | 0.2164253 | 0.1717786 | 0.2689084 |
| **PhA** | 1.250627 | 1.117936 | 1.532609 | 1.118314 | 0.7995989 | 0.6524821 | 0.8945054 |
| **Average daily intake of vegetables** | 1.065138 | 1.007336 | 1.578362 | 1.032055 | 0.9388451 | 0.6335683 | 0.9927172 |
| **Average daily intake of pork** | 1.248959 | 1.116758 | 1.530848 | 1.117569 | 0.8006666 | 0.6532329 | 0.8954491 |
| **Average daily intake of poultry** | 1.326421 | 1.172597 | 1.617338 | 1.151703 | 0.7539087 | 0.6183000 | 0.8528082 |
| **Low-density lipoprotein** | 1.117751 | 1.031441 | 1.440989 | 1.057237 | 0.8946540 | 0.6939677 | 0.9695173 |
