# Supplementary material for: Associations of dietary factors and early-life agricultural occupational background with body composition among older adults with type 2 diabetes in suburban Chengdu: A cross-sectional study
Source: Medicine (Baltimore). 2026 Jul 3;105(27):e49534. doi: 10.1097/MD.0000000000049534 (PMC13337032; doi:10.1097/MD.0000000000049534)
Supplement: Supplementary file 3 [file medi-105-e49534-s003.docx]

**Supplementary Table 3.** Univariate and multivariate analysis of influencing factors (SMI Logistic regression) in the non-agricultural group.

|  | **Univariable** | | | | | | **Multivariable** | | | | | |
| --- | --- | --- | --- | --- | --- | --- | --- | --- | --- | --- | --- | --- |
|  | **N** | **Event N** | **Estimate** | **SE** | **95% CI** | ***P*** | **N** | **Event N** | **Estimate** | **SE** | **95% CI** | ***P*** |
| **Sex** |  |  |  |  |  |  |  |  |  |  |  |  |
| Male | 123 | 47 | — | — | — |  |  |  |  |  |  |  |
| Female | 112 | 55 | 0.4448676560 | 0.2648776454 | -0.0742829894, 0.9640183013 | 0.093 |  |  |  |  |  |  |
| **Age** | 235 | 102 | 0.0580924908 | 0.0190836707 | 0.0206891834, 0.0954957981 | 0.002** | 235 | 102 | 0.0387469350 | 0.0294360816 | -0.0189467248, 0.0964405948 | 0.188 |
| **BMI** | 235 | 102 | -0.3833768065 | 0.0625957718 | -0.5060622649, -0.2606913482 | <0.001*** | 235 | 102 | -0.5642910156 | 0.1223209139 | -0.8040356014, -0.3245464299 | <0.001*** |
| **Systolic blood pressure** | 235 | 102 | -0.0063650419 | 0.0071310647 | -0.0203416720, 0.0076115882 | 0.372 |  |  |  |  |  |  |
| **Diastolic blood pressure** | 235 | 102 | -0.0145045257 | 0.0128363862 | -0.0396633804, 0.0106543289 | 0.258 |  |  |  |  |  |  |
| **WC** | 235 | 102 | -0.0607399032 | 0.0161993267 | -0.0924900000, -0.0289898063 | <0.001*** | 235 | 102 | 0.0555166771 | 0.0366420142 | -0.0163003510, 0.1273337052 | 0.130 |
| **HC** | 235 | 102 | -0.0777137944 | 0.0214149396 | -0.1196863046, -0.0357412841 | <0.001*** | 235 | 102 | 0.0188654158 | 0.0373918874 | -0.0544213369, 0.0921521684 | 0.614 |
| **duration of diabetes** | 235 | 102 | 0.0280793861 | 0.0182083029 | -0.0076082318, 0.0637670040 | 0.123 |  |  |  |  |  |  |
| **VFA** | 235 | 102 | -0.0016453751 | 0.0033687854 | -0.0082480732, 0.0049573230 | 0.625 |  |  |  |  |  |  |
| **Extracellular water ratio** | 235 | 102 | 57.0585652670 | 16.6171426029 | 24.4895642394, 89.6275662947 | <0.001*** | 235 | 102 | -179.1027043599 | 58.0032453793 | -292.7869762898, -65.4184324299 | 0.002** |
| **PhA** | 235 | 102 | -1.2821376351 | 0.2260609131 | -1.7252088831, -0.8390663871 | <0.001*** | 235 | 102 | -3.0168901782 | 0.7072414062 | -4.4030578627, -1.6307224937 | <0.001*** |
| **Average daily intake of rice** | 235 | 102 | -0.0026413541 | 0.0009813889 | -0.0045648410, -0.0007178672 | 0.007** | 235 | 102 | -0.0010225699 | 0.0015760853 | -0.0041116403, 0.0020665006 | 0.516 |
| **Average daily intake of flour** | 235 | 102 | -0.0067494398 | 0.0027084928 | -0.0120579881, -0.0014408916 | 0.013* | 235 | 102 | -0.0025722400 | 0.0039463427 | -0.0103069296, 0.0051624496 | 0.515 |
| **Average daily intake of other cereals** | 235 | 102 | -0.0009458997 | 0.0022059313 | -0.0052694456, 0.0033776462 | 0.668 |  |  |  |  |  |  |
| **Average daily intake of tubers** | 235 | 102 | -0.0079531535 | 0.0048106318 | -0.0173818186, 0.0014755116 | 0.098 |  |  |  |  |  |  |
| **Average daily intake of dairy products** | 235 | 102 | -0.0003718167 | 0.0011206230 | -0.0025681973, 0.0018245640 | 0.740 |  |  |  |  |  |  |
| **Average daily intake of eggs** | 235 | 102 | -0.5558586066 | 0.3087428912 | -1.1609835538, 0.0492663405 | 0.072 |  |  |  |  |  |  |
| **Average daily intake of dried beans** | 235 | 102 | -0.0339856597 | 0.0144757672 | -0.0623576421, -0.0056136773 | 0.019* | 235 | 102 | -0.0268326741 | 0.0161126281 | -0.0584128448, 0.0047474967 | 0.096 |
| **Average daily intake of soy products** | 235 | 102 | -0.0037527704 | 0.0048288994 | -0.0132172393, 0.0057116985 | 0.437 |  |  |  |  |  |  |
| **Average daily intake of vegetables** | 235 | 102 | -0.0000285992 | 0.0009445773 | -0.0018799367, 0.0018227384 | 0.976 |  |  |  |  |  |  |
| **Average daily intake of fruits** | 235 | 102 | -0.0000916550 | 0.0013927977 | -0.0028214883, 0.0026381782 | 0.948 |  |  |  |  |  |  |
| **Average daily intake of pork** | 235 | 102 | -0.0017223756 | 0.0014712941 | -0.0046060591, 0.0011613079 | 0.242 |  |  |  |  |  |  |
| **Average daily intake of poultry** | 235 | 102 | 0.0031409653 | 0.0063527702 | -0.0093102355, 0.0155921662 | 0.621 |  |  |  |  |  |  |
| **Average daily intake of beef and mutton** | 235 | 102 | -0.0315303957 | 0.0102778356 | -0.0516745833, -0.0113862081 | 0.002** | 235 | 102 | -0.0311804604 | 0.0114001226 | -0.0535242901, -0.0088366306 | 0.006** |
| **Average daily intake of aquatic products** | 235 | 102 | -0.0018882840 | 0.0068621227 | -0.0153377973, 0.0115612294 | 0.783 |  |  |  |  |  |  |
| **Hemoglobin** | 235 | 102 | 0.0103639241 | 0.0083123440 | -0.0059279707, 0.0266558189 | 0.212 |  |  |  |  |  |  |
| **Albumin** | 235 | 102 | 0.0137670037 | 0.0290236241 | -0.0431182543, 0.0706522616 | 0.635 |  |  |  |  |  |  |
| **Prealbumin** | 235 | 102 | -0.0008316267 | 0.0025107576 | -0.0057526211, 0.0040893677 | 0.740 |  |  |  |  |  |  |
| **Urea** | 235 | 102 | 0.0314229441 | 0.0367413672 | -0.0405888124, 0.1034347006 | 0.392 |  |  |  |  |  |  |
| **Creatinine** | 235 | 102 | -0.0054563195 | 0.0062316872 | -0.0176702019, 0.0067575629 | 0.381 |  |  |  |  |  |  |
| **Vitamin D level** | 235 | 102 | 0.0103435280 | 0.0059402661 | -0.0012991796, 0.0219862355 | 0.082 |  |  |  |  |  |  |
| **Total cholesterol** | 235 | 102 | -0.0597292912 | 0.1154357005 | -0.2859791067, 0.1665205244 | 0.605 |  |  |  |  |  |  |
| **Triglycerides** | 235 | 102 | 0.0028405276 | 0.1250518077 | -0.2422565116, 0.2479375668 | 0.982 |  |  |  |  |  |  |
| **High-density lipoprotein** | 235 | 102 | -0.1858637608 | 0.3853697887 | -0.9411746673, 0.5694471458 | 0.630 |  |  |  |  |  |  |
| **Low-density lipoprotein** | 235 | 102 | -0.2175077005 | 0.1682562998 | -0.5472839882, 0.1122685872 | 0.196 |  |  |  |  |  |  |
| **Alanine aminotransferase** | 235 | 102 | 0.0061205410 | 0.0067156827 | -0.0070419553, 0.0192830372 | 0.362 |  |  |  |  |  |  |
| **Aspartate aminotransferase** | 235 | 102 | 0.0018123249 | 0.0064591238 | -0.0108473250, 0.0144719749 | 0.779 |  |  |  |  |  |  |
| **HbA1c** | 235 | 102 | 0.0425715694 | 0.1299457046 | -0.2121173315, 0.2972604704 | 0.743 |  |  |  |  |  |  |
| **Fasting blood glucose** | 235 | 102 | -0.0412194693 | 0.0279927703 | -0.0960842910, 0.0136453524 | 0.141 |  |  |  |  |  |  |
| ^1^*p<0.05; **p<0.01; ***p<0.001 | | | | | | | | | | | | |
| Abbreviations: CI = Confidence Interval, OR = Odds Ratio, SE = Standard Error, NA | | | | | | | | | | | | |
