# Supplementary material for: Associations of dietary factors and early-life agricultural occupational background with body composition among older adults with type 2 diabetes in suburban Chengdu: A cross-sectional study
Source: Medicine (Baltimore). 2026 Jul 3;105(27):e49534. doi: 10.1097/MD.0000000000049534 (PMC13337032; doi:10.1097/MD.0000000000049534)
Supplement: Supplementary file 4 [file medi-105-e49534-s004.docx]

**Supplementary Table 4.** Variance Inflation Factor and Tolerance (SMI Logistic regression) in the non-agricultural group.

|  | VIF | VIF CI low | VIF CI high | SE factor | Tolerance | Tolerance CI low | Tolerance CI high |
| --- | --- | --- | --- | --- | --- | --- | --- |
| **Age** | 1.344355 | 1.190653 | 1.621971 | 1.159463 | 0.7438509 | 0.6165337 | 0.8398753 |
| **BMI** | 2.749703 | 2.283287 | 3.385640 | 1.658223 | 0.3636756 | 0.2953652 | 0.4379651 |
| **WC** | 3.058373 | 2.525355 | 3.777648 | 1.748820 | 0.3269713 | 0.2647150 | 0.3959840 |
| **HC** | 2.463530 | 2.059034 | 3.022523 | 1.569564 | 0.4059215 | 0.3308494 | 0.4856646 |
| **Extracellular water ratio** | 6.758192 | 5.430747 | 8.483339 | 2.599652 | 0.1479686 | 0.1178781 | 0.1841367 |
| **PhA** | 6.688861 | 5.376281 | 8.395123 | 2.586283 | 0.1495023 | 0.1191168 | 0.1860022 |
| **Average daily intake of rice** | 1.351313 | 1.195858 | 1.630154 | 1.162460 | 0.7400210 | 0.6134391 | 0.8362195 |
| **Average daily intake of wheat flour** | 1.095788 | 1.021379 | 1.429173 | 1.046799 | 0.9125850 | 0.6997054 | 0.9790682 |
| **Average daily intake of dried beans** | 1.054927 | 1.004807 | 1.627675 | 1.027096 | 0.9479329 | 0.6143731 | 0.9952164 |
| **Average daily intake of beef and mutton** | 1.103563 | 1.025405 | 1.422172 | 1.050506 | 0.9061555 | 0.7031497 | 0.9752243 |
