# Supplementary material for: Associations of dietary factors and early-life agricultural occupational background with body composition among older adults with type 2 diabetes in suburban Chengdu: A cross-sectional study
Source: Medicine (Baltimore). 2026 Jul 3;105(27):e49534. doi: 10.1097/MD.0000000000049534 (PMC13337032; doi:10.1097/MD.0000000000049534)
Supplement: Supplementary file 5 [file medi-105-e49534-s005.docx]

**Supplementary Table 5.** Univariate and multivariate analysis of influencing factors (PhA Linear regression) in the agricultural group

|  | **Univariable** | | | | | **Multivariable** | | | | |
| --- | --- | --- | --- | --- | --- | --- | --- | --- | --- | --- |
|  | **N** | **Estimate** | **SE** | **95% CI** | ***P*** | **N** | **Estimate** | **SE** | **95% CI** | ***P*** |
| **Sex** |  |  |  |  |  |  |  |  |  |  |
| Male | 42 | — | — | — |  | 42 | — | — | — |  |
| Female | 171 | -0.4025898079 | 0.1203654713 | -0.6385017965, -0.1666778192 | <0.001*** | 171 | 0.0399853477 | 0.1527478538 | -0.2593949445, 0.3393656400 | 0.794 |
| **Age** | 213 | -0.0421653786 | 0.0085547381 | -0.0589323571, -0.0253984001 | <0.001*** | 213 | -0.0288688131 | 0.0080750745 | -0.0446956683, -0.0130419579 | <0.001*** |
| **BMI** | 213 | 0.0532815586 | 0.0121958822 | 0.0293780687, 0.0771850485 | <0.001*** | 213 | 0.0038529777 | 0.0224785001 | -0.0402040728, 0.0479100282 | 0.864 |
| **household registration** |  |  |  |  |  |  |  |  |  |  |
| Urban | 13 | — | — | — |  |  |  |  |  |  |
| Rural | 200 | -0.1678846154 | 0.2049589307 | -0.5695967378, 0.2338275070 | 0.414 |  |  |  |  |  |
| **Systolic blood pressure** | 213 | -0.0027711984 | 0.0024232855 | -0.0075207508, 0.0019783540 | 0.254 |  |  |  |  |  |
| **Diastolic blood pressure** | 213 | 0.0050902020 | 0.0042277647 | -0.0031960645, 0.0133764684 | 0.230 |  |  |  |  |  |
| **WC** | 213 | 0.0185858886 | 0.0047241691 | 0.0093266873, 0.0278450900 | <0.001*** | 213 | 0.0025600890 | 0.0082130159 | -0.0135371263, 0.0186573043 | 0.756 |
| **HC** | 213 | 0.0211711212 | 0.0059016717 | 0.0096040574, 0.0327381851 | <0.001*** | 213 | -0.0038107400 | 0.0087494077 | -0.0209592640, 0.0133377840 | 0.664 |
| **SMI** | 213 | 0.4296917557 | 0.0468528872 | 0.3378617842, 0.5215217272 | <0.001*** | 213 | 0.3874417975 | 0.0832663517 | 0.2242427470, 0.5506408479 | <0.001*** |
| **duration of diabetes** | 213 | -0.0116343440 | 0.0067569233 | -0.0248776703, 0.0016089823 | 0.087 |  |  |  |  |  |
| **VFA** | 213 | -0.0000871078 | 0.0011545169 | -0.0023499192, 0.0021757037 | 0.940 |  |  |  |  |  |
| **Average daily intake of rice** | 213 | 0.0001726924 | 0.0003152030 | -0.0004450941, 0.0007904789 | 0.584 |  |  |  |  |  |
| **Average daily intake of flour** | 213 | 0.0013134137 | 0.0009436347 | -0.0005360763, 0.0031629038 | 0.165 |  |  |  |  |  |
| **Average daily intake of other cereals** | 213 | -0.0000435472 | 0.0012508969 | -0.0024952602, 0.0024081657 | 0.972 |  |  |  |  |  |
| **Average daily intake of tubers** | 213 | 0.0003768969 | 0.0015747103 | -0.0027094786, 0.0034632725 | 0.811 |  |  |  |  |  |
| **Average daily intake of dairy products** | 213 | -0.0002315920 | 0.0004085976 | -0.0010324285, 0.0005692445 | 0.571 |  |  |  |  |  |
| **Average daily intake of eggs** | 213 | 0.0228461081 | 0.0916694593 | -0.1568227306, 0.2025149467 | 0.803 |  |  |  |  |  |
| **Average daily intake of dried beans** | 213 | -0.0009669241 | 0.0042160979 | -0.0092303242, 0.0072964760 | 0.819 |  |  |  |  |  |
| **Average daily intake of soy products** | 213 | 0.0025637688 | 0.0026983853 | -0.0027249692, 0.0078525069 | 0.343 |  |  |  |  |  |
| **Average daily intake of vegetables** | 213 | 0.0004651087 | 0.0003028187 | -0.0001284049, 0.0010586224 | 0.126 |  |  |  |  |  |
| **Average daily intake of fruits** | 213 | 0.0002762664 | 0.0006355941 | -0.0009694750, 0.0015220079 | 0.664 |  |  |  |  |  |
| **Average daily intake of pork** | 213 | 0.0018671380 | 0.0006573397 | 0.0005787759, 0.0031555001 | 0.005** | 213 | 0.0004097244 | 0.0006501290 | -0.0008645050, 0.0016839538 | 0.529 |
| **Average daily intake of poultry** | 213 | 0.0069548209 | 0.0033290406 | 0.0004300212, 0.0134796207 | 0.038* | 213 | 0.0002600889 | 0.0031682900 | -0.0059496454, 0.0064698233 | 0.935 |
| **Average daily intake of beef and mutton** | 213 | 0.0007556516 | 0.0032719494 | -0.0056572513, 0.0071685545 | 0.818 |  |  |  |  |  |
| **Average daily intake of aquatic products** | 213 | 0.0028171653 | 0.0028129516 | -0.0026961186, 0.0083304491 | 0.318 |  |  |  |  |  |
| **Hemoglobin** | 213 | -0.0007025526 | 0.0006421388 | -0.0019611216, 0.0005560164 | 0.275 |  |  |  |  |  |
| **Albumin** | 213 | 0.0087313182 | 0.0097938769 | -0.0104643279, 0.0279269643 | 0.374 |  |  |  |  |  |
| **Prealbumin** | 213 | -0.0001263002 | 0.0008803054 | -0.0018516671, 0.0015990667 | 0.886 |  |  |  |  |  |
| **Urea** | 213 | -0.0039236307 | 0.0200192412 | -0.0431606225, 0.0353133610 | 0.845 |  |  |  |  |  |
| **Creatinine** | 213 | -0.0006724655 | 0.0019676740 | -0.0045290358, 0.0031841047 | 0.733 |  |  |  |  |  |
| **Vitamin D level** | 213 | -0.0012057616 | 0.0018857905 | -0.0049018431, 0.0024903198 | 0.523 |  |  |  |  |  |
| **Total cholesterol** | 213 | 0.0155171160 | 0.0403495276 | -0.0635665050, 0.0946007369 | 0.701 |  |  |  |  |  |
| **Triglycerides** | 213 | 0.0324459102 | 0.0175929768 | -0.0020356908, 0.0669275112 | 0.067 |  |  |  |  |  |
| **High-density lipoprotein** | 213 | 0.1004188792 | 0.1117288963 | -0.1185657336, 0.3194034921 | 0.370 |  |  |  |  |  |
| **Low-density lipoprotein** | 213 | -0.0080799696 | 0.0591633906 | -0.1240380844, 0.1078781452 | 0.892 |  |  |  |  |  |
| **Alanine aminotransferase** | 213 | -0.0008794682 | 0.0023814535 | -0.0055470312, 0.0037880949 | 0.712 |  |  |  |  |  |
| **Aspartate aminotransferase** | 213 | 0.0008563448 | 0.0048064701 | -0.0085641635, 0.0102768532 | 0.859 |  |  |  |  |  |
| **HbA1c** | 213 | -0.1247340305 | 0.6020033808 | -1.3046389754, 1.0551709143 | 0.836 |  |  |  |  |  |
| **Fasting blood glucose** | 213 | -0.0082699681 | 0.0107239172 | -0.0292884595, 0.0127485233 | 0.441 |  |  |  |  |  |
| ^1^*p<0.05; **p<0.01; ***p<0.001 | | | | | | | | | | |
| Abbreviations: CI = Confidence Interval, SE = Standard Error, NA | | | | | | | | | | |
