# Supplementary material for: Associations of dietary factors and early-life agricultural occupational background with body composition among older adults with type 2 diabetes in suburban Chengdu: A cross-sectional study
Source: Medicine (Baltimore). 2026 Jul 3;105(27):e49534. doi: 10.1097/MD.0000000000049534 (PMC13337032; doi:10.1097/MD.0000000000049534)
Supplement: Supplementary file 6 [file medi-105-e49534-s006.docx]

**Supplementary Table 6.** Variance Inflation Factor and Tolerance (PhA Linear regression) in the agricultural group

|  | VIF | VIF CI low | VIF CI high | SE factor | Tolerance | Tolerance CI low | Tolerance CI high |
| --- | --- | --- | --- | --- | --- | --- | --- |
| **Sex** | 2.228790 | 1.859330 | 2.757096 | 1.492913 | 0.4486739 | 0.3627004 | 0.5378282 |
| **Age** | 1.164423 | 1.058865 | 1.459271 | 1.079084 | 0.8587946 | 0.6852736 | 0.9444078 |
| **BMI** | 4.540031 | 3.648607 | 5.731475 | 2.130735 | 0.2202628 | 0.1744751 | 0.2740772 |
| **WC** | 4.103649 | 3.310419 | 5.169217 | 2.025747 | 0.2436856 | 0.1934529 | 0.3020766 |
| **HC** | 3.018947 | 2.470239 | 3.772439 | 1.737512 | 0.3312413 | 0.2650805 | 0.4048192 |
| **SMI** | 3.290986 | 2.680861 | 4.122577 | 1.814108 | 0.3038603 | 0.2425668 | 0.3730144 |
| **Average daily intake of pork** | 1.373036 | 1.205771 | 1.676268 | 1.171766 | 0.7283129 | 0.5965635 | 0.8293451 |
| **Average daily intake of poultry** | 1.293241 | 1.147432 | 1.583253 | 1.137207 | 0.7732512 | 0.6316109 | 0.8715113 |
