# Supplementary material for: Associations of dietary factors and early-life agricultural occupational background with body composition among older adults with type 2 diabetes in suburban Chengdu: A cross-sectional study
Source: Medicine (Baltimore). 2026 Jul 3;105(27):e49534. doi: 10.1097/MD.0000000000049534 (PMC13337032; doi:10.1097/MD.0000000000049534)
Supplement: Supplementary file 7 [file medi-105-e49534-s007.docx]

**Supplementary Table 7.** Univariate and multivariate analysis of influencing factors (PhA Linear regression) in the non-agricultural group

|  | **Univariable** | | | | | **Multivariable** | | | | |
| --- | --- | --- | --- | --- | --- | --- | --- | --- | --- | --- |
|  | **N** | **Estimate** | **SE** | **95% CI** | ***P*** | **N** | **Estimate** | **SE** | **95% CI** | ***P*** |
| **Sex** |  |  |  |  |  |  |  |  |  |  |
| Male | 123 | — | — | — |  | 123 | — | — | — |  |
| Female | 112 | -0.5980545877 | 0.0906574565 | -0.7757399373, -0.4203692381 | <0.001*** | 112 | 0.0691893866 | 0.1005801088 | -0.1279440042, 0.2663227773 | 0.492 |
| **Age** | 235 | -0.0511755018 | 0.0060223119 | -0.0629790162, -0.0393719875 | <0.001*** | 235 | -0.0276733546 | 0.0048674414 | -0.0372133644, -0.0181333448 | <0.001*** |
| **BMI** | 235 | 0.0375826080 | 0.0159879844 | 0.0062467343, 0.0689184816 | 0.020* | 235 | 0.1258516005 | 0.0255263361 | 0.0758209011, 0.1758822998 | <0.001*** |
| **household registration** |  |  |  |  |  |  |  |  |  |  |
| Urban | 148 | — | — | — |  | 148 | — | — | — |  |
| Rural | 87 | 0.4207983846 | 0.0983648400 | 0.2280068409, 0.6135899283 | <0.001*** | 87 | -0.0004219410 | 0.0720305103 | -0.1415991470, 0.1407552649 | 0.995 |
| **Systolic blood pressure** | 235 | -0.0027951337 | 0.0026480303 | -0.0079851778, 0.0023949104 | 0.292 |  |  |  |  |  |
| **Diastolic blood pressure** | 235 | 0.0066729562 | 0.0047527302 | -0.0026422239, 0.0159881363 | 0.162 |  |  |  |  |  |
| **WC** | 235 | 0.0029500098 | 0.0053345476 | -0.0075055115, 0.0134055310 | 0.581 |  |  |  |  |  |
| **HC** | 235 | 0.0032158837 | 0.0064988299 | -0.0095215889, 0.0159533564 | 0.621 |  |  |  |  |  |
| **SMI** | 235 | 0.4528817142 | 0.0418076589 | 0.3709402086, 0.5348232199 | <0.001*** | 235 | 0.1223501204 | 0.0742234862 | -0.0231252393, 0.2678254801 | 0.101 |
| **duration of diabetes** | 235 | -0.0266909118 | 0.0065149086 | -0.0394598980, -0.0139219255 | <0.001*** | 235 | -0.0161208428 | 0.0043556851 | -0.0246578287, -0.0075838568 | <0.001*** |
| **VFA** | 235 | -0.0065365669 | 0.0011827821 | -0.0088547772, -0.0042183565 | <0.001*** | 235 | -0.0114713574 | 0.0015761387 | -0.0145605325, -0.0083821823 | <0.001*** |
| **Average daily intake of rice** | 235 | 0.0020678473 | 0.0003062836 | 0.0014675425, 0.0026681521 | <0.001*** | 235 | 0.0008717982 | 0.0002600131 | 0.0003621818, 0.0013814145 | <0.001*** |
| **Average daily intake of flour** | 235 | 0.0018242437 | 0.0009192562 | 0.0000225347, 0.0036259528 | 0.048* | 235 | -0.0012731213 | 0.0006364109 | -0.0025204637, -0.0000257789 | 0.047* |
| **Average daily intake of other cereals** | 235 | -0.0008504302 | 0.0008142951 | -0.0024464194, 0.0007455589 | 0.297 |  |  |  |  |  |
| **Average daily intake of tubers** | 235 | -0.0019555037 | 0.0011009947 | -0.0041134137, 0.0002024063 | 0.077 |  |  |  |  |  |
| **Average daily intake of dairy products** | 235 | -0.0006252321 | 0.0004175660 | -0.0014436463, 0.0001931822 | 0.136 |  |  |  |  |  |
| **Average daily intake of eggs** | 235 | -0.0361069813 | 0.1104366269 | -0.2525587926, 0.1803448299 | 0.744 |  |  |  |  |  |
| **Average daily intake of dried beans** | 235 | 0.0023909401 | 0.0032015867 | -0.0038840546, 0.0086659348 | 0.456 |  |  |  |  |  |
| **Average daily intake of soy products** | 235 | -0.0004171981 | 0.0016740018 | -0.0036981812, 0.0028637851 | 0.803 |  |  |  |  |  |
| **Average daily intake of vegetables** | 235 | 0.0006049540 | 0.0003517393 | -0.0000844424, 0.0012943505 | 0.087 |  |  |  |  |  |
| **Average daily intake of fruits** | 235 | -0.0004742622 | 0.0005203948 | -0.0014942173, 0.0005456930 | 0.363 |  |  |  |  |  |
| **Average daily intake of pork** | 235 | 0.0026952789 | 0.0005052360 | 0.0017050346, 0.0036855232 | <0.001*** | 235 | 0.0008809453 | 0.0003870486 | 0.0001223439, 0.0016395467 | 0.024* |
| **Average daily intake of poultry** | 235 | 0.0029801992 | 0.0023773952 | -0.0016794098, 0.0076398081 | 0.211 |  |  |  |  |  |
| **Average daily intake of beef and mutton** | 235 | 0.0007341896 | 0.0025952886 | -0.0043524827, 0.0058208618 | 0.778 |  |  |  |  |  |
| **Average daily intake of aquatic products** | 235 | 0.0003319278 | 0.0025492979 | -0.0046646043, 0.0053284599 | 0.897 |  |  |  |  |  |
| **Hemoglobin** | 235 | -0.0047452053 | 0.0030478258 | -0.0107188341, 0.0012284234 | 0.121 |  |  |  |  |  |
| **Albumin** | 235 | 0.0060305372 | 0.0108600804 | -0.0152548292, 0.0273159036 | 0.579 |  |  |  |  |  |
| **Prealbumin** | 235 | 0.0005205508 | 0.0009383215 | -0.0013185256, 0.0023596272 | 0.580 |  |  |  |  |  |
| **Urea** | 235 | 0.0144480047 | 0.0112925249 | -0.0076849374, 0.0365809469 | 0.202 |  |  |  |  |  |
| **Creatinine** | 235 | 0.0043183266 | 0.0022373448 | -0.0000667885, 0.0087034418 | 0.055 |  |  |  |  |  |
| **Vitamin D level** | 235 | -0.0052322949 | 0.0021628641 | -0.0094714306, -0.0009931592 | 0.016* | 235 | -0.0027977581 | 0.0013976991 | -0.0055371980, -0.0000583181 | 0.047* |
| **Total cholesterol** | 235 | -0.0062469227 | 0.0431241714 | -0.0907687454, 0.0782749000 | 0.885 |  |  |  |  |  |
| **Triglycerides** | 235 | 0.0293051590 | 0.0468486501 | -0.0625165080, 0.1211268260 | 0.532 |  |  |  |  |  |
| **High-density lipoprotein** | 235 | -0.0733809266 | 0.1430631698 | -0.3537795869, 0.2070177337 | 0.608 |  |  |  |  |  |
| **Low-density lipoprotein** | 235 | 0.0186367494 | 0.0620704090 | -0.1030190168, 0.1402925155 | 0.764 |  |  |  |  |  |
| **Alanine aminotransferase** | 235 | 0.0003026572 | 0.0024858700 | -0.0045695585, 0.0051748729 | 0.903 |  |  |  |  |  |
| **Aspartate aminotransferase** | 235 | -0.0042253250 | 0.0024193191 | -0.0089671033, 0.0005164532 | 0.082 |  |  |  |  |  |
| **HbA1c** | 235 | 0.0819264030 | 0.0485745495 | -0.0132779645, 0.1771307705 | 0.093 |  |  |  |  |  |
| **Fasting blood glucose** | 235 | -0.0017801073 | 0.0095618194 | -0.0205209289, 0.0169607143 | 0.852 |  |  |  |  |  |
| ^1^*p<0.05; **p<0.01; ***p<0.001 | | | | | | | | | | |
| Abbreviations: CI = Confidence Interval, SE = Standard Error, NA | | | | | | | | | | |
