# Supplementary material for: Associations of dietary factors and early-life agricultural occupational background with body composition among older adults with type 2 diabetes in suburban Chengdu: A cross-sectional study
Source: Medicine (Baltimore). 2026 Jul 3;105(27):e49534. doi: 10.1097/MD.0000000000049534 (PMC13337032; doi:10.1097/MD.0000000000049534)
Supplement: Supplementary file 8 [file medi-105-e49534-s008.docx]

**Supplementary Table 8.** Variance Inflation Factor and Tolerance (PhA Linear regression) in the non-agricultural group

|  | VIF | VIF CI low | VIF CI high | SE factor | Tolerance | Tolerance CI low | Tolerance CI high |
| --- | --- | --- | --- | --- | --- | --- | --- |
| **Sex** | 2.666582 | 2.219843 | 3.276927 | 1.632967 | 0.3750120 | 0.3051640 | 0.4504822 |
| **Age** | 1.282148 | 1.145041 | 1.548860 | 1.132320 | 0.7799412 | 0.6456361 | 0.8733308 |
| **BMI** | 6.402011 | 5.155848 | 8.021844 | 2.530219 | 0.1562009 | 0.1246596 | 0.1939545 |
| **household registration** | 1.278267 | 1.142200 | 1.544531 | 1.130605 | 0.7823093 | 0.6474457 | 0.8755034 |
| **SMI** | 5.389387 | 4.359558 | 6.734896 | 2.321505 | 0.1855499 | 0.1484804 | 0.2293811 |
| **duration of diabetes** | 1.071995 | 1.010692 | 1.484771 | 1.035372 | 0.9328403 | 0.6735046 | 0.9894209 |
| **VFA** | 4.036384 | 3.295850 | 5.015779 | 2.009075 | 0.2477465 | 0.1993708 | 0.3034119 |
| **Average daily intake of rice** | 1.549718 | 1.347596 | 1.869371 | 1.244877 | 0.6452787 | 0.5349392 | 0.7420622 |
| **Average daily intake of flour** | 1.211795 | 1.094529 | 1.474532 | 1.100816 | 0.8252222 | 0.6781812 | 0.9136350 |
| **Average daily intake of pork** | 1.344625 | 1.191361 | 1.620640 | 1.159579 | 0.7437020 | 0.6170400 | 0.8393765 |
| **Vitamin D level** | 1.047373 | 1.002958 | 1.758664 | 1.023413 | 0.9547693 | 0.5686135 | 0.9970506 |
