# Supplementary material for: Associations of dietary factors and early-life agricultural occupational background with body composition among older adults with type 2 diabetes in suburban Chengdu: A cross-sectional study
Source: Medicine (Baltimore). 2026 Jul 3;105(27):e49534. doi: 10.1097/MD.0000000000049534 (PMC13337032; doi:10.1097/MD.0000000000049534)
Supplement: Supplementary file 9 [file medi-105-e49534-s009.docx]

**Supplementary Table 9.** Univariate and multivariate analysis of influencing factors (PhA Logistic regression) in the agricultural group

| **Characteristic** | **Univariable** | | | | | **Multivariable** | | | | |
| --- | --- | --- | --- | --- | --- | --- | --- | --- | --- | --- |
|  | **N** | **Event N** | **OR** | **95% CI** | ***P*** | **N** | **Event N** | **OR** | **95% CI** | ***P*** |
| **Sex** |  |  |  |  |  |  |  |  |  |  |
| Male | 42 | 15 | — | — |  |  |  |  |  |  |
| Female | 171 | 45 | 0.643 | 0.314, 1.317 | 0.227 |  |  |  |  |  |
| **Age** | 213 | 60 | 1.152 | 1.079, 1.230 | <0.001*** | 213 | 60 | 1.163 | 1.058, 1.277 | 0.002** |
| **BMI** | 213 | 60 | 0.738 | 0.659, 0.826 | <0.001*** | 213 | 60 | 0.753 | 0.520, 1.090 | 0.133 |
| **Systolic blood pressure** | 213 | 60 | 1.002 | 0.987, 1.016 | 0.835 |  |  |  |  |  |
| **Diastolic blood pressure** | 213 | 60 | 0.978 | 0.952, 1.004 | 0.099 |  |  |  |  |  |
| **WC** | 213 | 60 | 0.929 | 0.897, 0.962 | <0.001*** | 213 | 60 | 1.030 | 0.936, 1.134 | 0.541 |
| **HC** | 213 | 60 | 0.928 | 0.889, 0.968 | <0.001*** | 213 | 60 | 1.010 | 0.919, 1.111 | 0.831 |
| **SMI** | 213 | 60 | 0.297 | 0.189, 0.467 | <0.001*** | 213 | 60 | 0.595 | 0.159, 2.227 | 0.441 |
| **duration of diabetes** | 213 | 60 | 1.005 | 0.964, 1.047 | 0.818 |  |  |  |  |  |
| **Body fat** | 213 | 60 | 0.886 | 0.839, 0.935 | <0.001*** | 213 | 60 | 0.463 | 0.289, 0.744 | 0.001** |
| **Body fat percentage** | 213 | 60 | 0.943 | 0.907, 0.979 | 0.002** | 213 | 60 | 0.739 | 0.586, 0.931 | 0.010* |
| **VFA** | 213 | 60 | 0.991 | 0.983, 0.998 | 0.014* | 213 | 60 | 1.188 | 1.106, 1.275 | <0.001*** |
| **Average daily intake of rice** | 213 | 60 | 0.999 | 0.997, 1.001 | 0.561 |  |  |  |  |  |
| **Average daily intake of flour** | 213 | 60 | 0.997 | 0.991, 1.003 | 0.401 |  |  |  |  |  |
| **Average daily intake of other cereals** | 213 | 60 | 1.000 | 0.993, 1.008 | 0.980 |  |  |  |  |  |
| **Average daily intake of tubers** | 213 | 60 | 0.996 | 0.986, 1.006 | 0.451 |  |  |  |  |  |
| **Average daily intake of dairy products** | 213 | 60 | 0.999 | 0.997, 1.002 | 0.607 |  |  |  |  |  |
| **Average daily intake of eggs** | 213 | 60 | 0.868 | 0.494, 1.526 | 0.624 |  |  |  |  |  |
| **Average daily intake of dried beans** | 213 | 60 | 0.995 | 0.968, 1.023 | 0.724 |  |  |  |  |  |
| **Average daily intake of soy products** | 213 | 60 | 0.997 | 0.980, 1.014 | 0.732 |  |  |  |  |  |
| **Average daily intake of vegetables** | 213 | 60 | 0.997 | 0.995, 0.999 | 0.014* | 213 | 60 | 0.998 | 0.996, 1.001 | 0.306 |
| **Average daily intake of fruits** | 213 | 60 | 0.998 | 0.994, 1.002 | 0.379 |  |  |  |  |  |
| **Average daily intake of pork** | 213 | 60 | 0.996 | 0.992, 1.001 | 0.102 |  |  |  |  |  |
| **Average daily intake of poultry** | 213 | 60 | 0.971 | 0.942, 1.001 | 0.061 |  |  |  |  |  |
| **Average daily intake of beef and mutton** | 213 | 60 | 0.985 | 0.946, 1.025 | 0.454 |  |  |  |  |  |
| **Average daily intake of aquatic products** | 213 | 60 | 0.984 | 0.957, 1.012 | 0.270 |  |  |  |  |  |
| **Hemoglobin** | 213 | 60 | 1.003 | 0.997, 1.009 | 0.325 |  |  |  |  |  |
| **Albumin** | 213 | 60 | 0.959 | 0.901, 1.020 | 0.187 |  |  |  |  |  |
| **Prealbumin** | 213 | 60 | 1.002 | 0.996, 1.007 | 0.530 |  |  |  |  |  |
| **Urea** | 213 | 60 | 0.972 | 0.858, 1.102 | 0.662 |  |  |  |  |  |
| **Creatinine** | 213 | 60 | 1.008 | 0.996, 1.019 | 0.184 |  |  |  |  |  |
| **Vitamin D level** | 213 | 60 | 1.008 | 0.997, 1.019 | 0.162 |  |  |  |  |  |
| **Total cholesterol** | 213 | 60 | 0.909 | 0.708, 1.168 | 0.457 |  |  |  |  |  |
| **Triglycerides** | 213 | 60 | 0.910 | 0.757, 1.093 | 0.312 |  |  |  |  |  |
| **High-density lipoprotein** | 213 | 60 | 0.598 | 0.287, 1.245 | 0.170 |  |  |  |  |  |
| **Low-density lipoprotein** | 213 | 60 | 0.977 | 0.681, 1.401 | 0.898 |  |  |  |  |  |
| **Alanine aminotransferase** | 213 | 60 | 1.008 | 0.994, 1.022 | 0.256 |  |  |  |  |  |
| **Aspartate aminotransferase** | 213 | 60 | 1.004 | 0.975, 1.033 | 0.794 |  |  |  |  |  |
| **HbA1c** | 213 | 60 | 1.604 | 0.054, 47.572 | 0.785 |  |  |  |  |  |
| **Fasting blood glucose** | 213 | 60 | 0.993 | 0.929, 1.060 | 0.824 |  |  |  |  |  |

1*p<0.05; **p<0.01; ***p<0.001

Abbreviations: CI = Confidence Interval, OR = Odds Ratio

Null deviance = 253; Null df = 212; Log-likelihood = -70.2; AIC = 160; BIC = 194; Deviance = 140; Residual df = 203; No. Obs. = 213
