# Supplementary material for: Associations of dietary factors and early-life agricultural occupational background with body composition among older adults with type 2 diabetes in suburban Chengdu: A cross-sectional study
Source: Medicine (Baltimore). 2026 Jul 3;105(27):e49534. doi: 10.1097/MD.0000000000049534 (PMC13337032; doi:10.1097/MD.0000000000049534)
Supplement: Supplementary file 10 [file medi-105-e49534-s010.docx]

**Supplementary Table 10.** Variance Inflation Factor and Tolerance (PhA Logistic regression) in the agricultural group

| Term | VIF | VIF CI low | VIF CI high | SE fator | Tolerance | Tolerance CI low | Tolerance CI high |
| --- | --- | --- | --- | --- | --- | --- | --- |
| **Age** | 1.222340 | 1.097752 | 1.505719 | 1.105595 | 0.81810287 | 0.66413459 | 0.91095238 |
| **BMI** | 8.154148 | 6.457922 | 10.377531 | 2.855547 | 0.12263697 | 0.09636203 | 0.15484858 |
| **WC** | 4.028394 | 3.255393 | 5.066330 | 2.007086 | 0.24823786 | 0.19738153 | 0.30718258 |
| **HC** | 2.809127 | 2.309951 | 3.498520 | 1.676045 | 0.35598252 | 0.28583517 | 0.43290956 |
| **SMI** | 6.058987 | 4.831363 | 7.679959 | 2.461501 | 0.16504410 | 0.13020903 | 0.20698094 |
| **Body fat** | 50.125555 | 39.048395 | 64.427645 | 7.079940 | 0.01994990 | 0.01552129 | 0.02560925 |
| **Body fat percentage** | 19.031262 | 14.903589 | 24.384353 | 4.362484 | 0.05254512 | 0.04100991 | 0.06709793 |
| **VFA** | 47.781149 | 37.227948 | 61.408498 | 6.912391 | 0.02092876 | 0.01628439 | 0.02686154 |
| **Average daily intake of vegetables** | 1.125761 | 1.035722 | 1.442744 | 1.061019 | 0.88828788 | 0.69312389 | 0.96550966 |
