# Supplementary material for: Associations of dietary factors and early-life agricultural occupational background with body composition among older adults with type 2 diabetes in suburban Chengdu: A cross-sectional study
Source: Medicine (Baltimore). 2026 Jul 3;105(27):e49534. doi: 10.1097/MD.0000000000049534 (PMC13337032; doi:10.1097/MD.0000000000049534)
Supplement: Supplementary file 11 [file medi-105-e49534-s011.docx]

**Supplementary Table 11.** Univariate and multivariate analysis of influencing factors (PhA Logistic regression) in the non-agricultural group

| **Characteristic** | **Univariable** | | | | | | | **Multivariable** | | | | | | | | | |
| --- | --- | --- | --- | --- | --- | --- | --- | --- | --- | --- | --- | --- | --- | --- | --- | --- | --- |
|  | **N** | **Event N** | **OR** | **95% CI** | | ***P*** | | **N** | | **Event N** | | **OR** | | **95% CI** | | ***P*** | |
| **Sex** |  |  |  |  |  | |  | |  | |  | |  | |  | |  |
| Male | 123 | 26 | — | — |  | |  | |  | |  | |  | |  | |  |
| Female | 112 | 28 | 1.244 | 0.677, 2.285 | 0.483 | |  | |  | |  | |  | |  | |  |
| **Age** | 235 | 54 | 1.138 | 1.086, 1.193 | <0.001*** | | 235 | | 54 | | 1.113 | | 1.048, 1.181 | | <0.001*** | |  |
| **BMI** | 235 | 54 | 0.870 | 0.779, 0.970 | 0.013* | | 235 | | 54 | | 0.400 | | 0.266, 0.602 | | <0.001*** | |  |
| **Systolic blood pressure** | 235 | 54 | 1.002 | 0.986, 1.018 | 0.830 | |  | |  | |  | |  | |  | |  |
| **Diastolic blood pressure** | 235 | 54 | 0.982 | 0.953, 1.012 | 0.230 | |  | |  | |  | |  | |  | |  |
| **WC** | 235 | 54 | 0.987 | 0.955, 1.020 | 0.434 | |  | |  | |  | |  | |  | |  |
| **HC** | 235 | 54 | 0.975 | 0.937, 1.014 | 0.201 | |  | |  | |  | |  | |  | |  |
| **SMI** | 235 | 54 | 0.365 | 0.246, 0.542 | <0.001*** | | 235 | | 54 | | 1.863 | | 0.883, 3.928 | | 0.102 | |  |
| **duration of diabetes** | 235 | 54 | 1.083 | 1.038, 1.130 | <0.001*** | | 235 | | 54 | | 1.076 | | 1.016, 1.140 | | 0.012* | |  |
| **Body fat** | 235 | 54 | 1.007 | 0.959, 1.058 | 0.768 | |  | |  | |  | |  | |  | |  |
| **Body fat percentage** | 235 | 54 | 1.042 | 1.000, 1.086 | 0.051 | |  | |  | |  | |  | |  | |  |
| **VFA** | 235 | 54 | 1.010 | 1.003, 1.018 | 0.008** | | 235 | | 54 | | 1.065 | | 1.037, 1.093 | | <0.001*** | |  |
| **Average daily intake of rice** | 235 | 54 | 0.994 | 0.991, 0.997 | <0.001*** | | 235 | | 54 | | 0.995 | | 0.990, 0.999 | | 0.016* | |  |
| **Average daily intake of flour** | 235 | 54 | 0.997 | 0.992, 1.003 | 0.411 | |  | |  | |  | |  | |  | |  |
| **Average daily intake of other cereals** | 235 | 54 | 1.002 | 0.997, 1.007 | 0.444 | |  | |  | |  | |  | |  | |  |
| **Average daily intake of tubers** | 235 | 54 | 1.003 | 0.997, 1.009 | 0.362 | |  | |  | |  | |  | |  | |  |
| **Average daily intake of dairy products** | 235 | 54 | 1.001 | 0.999, 1.004 | 0.336 | |  | |  | |  | |  | |  | |  |
| **Average daily intake of eggs** | 235 | 54 | 0.974 | 0.493, 1.927 | 0.941 | |  | |  | |  | |  | |  | |  |
| **Average daily intake of dried beans** | 235 | 54 | 0.990 | 0.967, 1.014 | 0.429 | |  | |  | |  | |  | |  | |  |
| **Average daily intake of soy products** | 235 | 54 | 1.001 | 0.991, 1.011 | 0.895 | |  | |  | |  | |  | |  | |  |
| **Average daily intake of vegetables** | 235 | 54 | 0.999 | 0.997, 1.001 | 0.282 | |  | |  | |  | |  | |  | |  |
| **Average daily intake of fruits** | 235 | 54 | 1.002 | 0.999, 1.005 | 0.310 | |  | |  | |  | |  | |  | |  |
| **Average daily intake of pork** | 235 | 54 | 0.997 | 0.993, 1.000 | 0.087 | |  | |  | |  | |  | |  | |  |
| **Average daily intake of poultry** | 235 | 54 | 1.000 | 0.985, 1.015 | 0.990 | |  | |  | |  | |  | |  | |  |
| **Average daily intake of beef and mutton** | 235 | 54 | 0.992 | 0.974, 1.010 | 0.389 | |  | |  | |  | |  | |  | |  |
| **Average daily intake of aquatic products** | 235 | 54 | 0.997 | 0.981, 1.014 | 0.718 | |  | |  | |  | |  | |  | |  |
| **Hemoglobin** | 235 | 54 | 1.023 | 1.002, 1.044 | 0.029* | | 235 | | 54 | | 1.013 | | 0.986, 1.042 | | 0.341 | |  |
| **Albumin** | 235 | 54 | 0.956 | 0.892, 1.024 | 0.201 | |  | |  | |  | |  | |  | |  |
| **Prealbumin** | 235 | 54 | 0.998 | 0.992, 1.004 | 0.471 | |  | |  | |  | |  | |  | |  |
| **Urea** | 235 | 54 | 0.844 | 0.717, 0.994 | 0.042* | | 235 | | 54 | | 0.893 | | 0.715, 1.116 | | 0.321 | |  |
| **Creatinine** | 235 | 54 | 0.975 | 0.956, 0.993 | 0.008** | | 235 | | 54 | | 0.969 | | 0.943, 0.996 | | 0.022* | |  |
| **Vitamin D level** | 235 | 54 | 1.011 | 0.998, 1.024 | 0.090 | |  | |  | |  | |  | |  | |  |
| **Total cholesterol** | 235 | 54 | 1.064 | 0.817, 1.387 | 0.644 | |  | |  | |  | |  | |  | |  |
| **Triglycerides** | 235 | 54 | 0.782 | 0.552, 1.108 | 0.166 | |  | |  | |  | |  | |  | |  |
| **High-density lipoprotein** | 235 | 54 | 1.322 | 0.562, 3.113 | 0.522 | |  | |  | |  | |  | |  | |  |
| **Low-density lipoprotein** | 235 | 54 | 0.947 | 0.644, 1.390 | 0.780 | |  | |  | |  | |  | |  | |  |
| **Alanine aminotransferase** | 235 | 54 | 1.002 | 0.987, 1.017 | 0.827 | |  | |  | |  | |  | |  | |  |
| **Aspartate aminotransferase** | 235 | 54 | 1.017 | 0.999, 1.034 | 0.057 | |  | |  | |  | |  | |  | |  |
| **HbA1c** | 235 | 54 | 0.786 | 0.326, 1.897 | 0.592 | |  | |  | |  | |  | |  | |  |
| **Fasting blood glucose** | 235 | 54 | 0.969 | 0.907, 1.036 | 0.359 | |  | |  | |  | |  | |  | |  |
| ^1^*p<0.05; **p<0.01; ***p<0.001 | | | | | | | | | | | | | | | | |  |
| Abbreviations: CI = Confidence Interval, OR = Odds Ratio | | | | | | | | | | | | | | | | |  |
| Null deviance = 253; Null df = 234; Log-likelihood = -73.9; AIC = 170; BIC = 208; Deviance = 148; Residual df = 224; No. Obs. = 235 | | | | | | | | | | | | | | | | |  |
