# Supplementary material for: Associations of dietary factors and early-life agricultural occupational background with body composition among older adults with type 2 diabetes in suburban Chengdu: A cross-sectional study
Source: Medicine (Baltimore). 2026 Jul 3;105(27):e49534. doi: 10.1097/MD.0000000000049534 (PMC13337032; doi:10.1097/MD.0000000000049534)
Supplement: Supplementary file 12 [file medi-105-e49534-s012.docx]

**Supplementary Table 12.** Variance Inflation Factor and Tolerance (PhA Logistic regression) in the non-agricultural group

| Term | VIF | VIF CI low | VIF CI high | SE factor | Tolerance | Tolerance CI low | Tolerance CI high |
| --- | --- | --- | --- | --- | --- | --- | --- |
| **Age** | 1.1310 | 1.041046 | 1.418490 | 1.063515 | 0.8841239 | 0.70497481 | 0.9605719 |
| **BMI** | 8.217927 | 6.577533 | 10.340773 | 2.866693 | 0.1216852 | 0.09670457 | 0.1520327 |
| **SMI** | 2.430196 | 2.032928 | 2.980255 | 1.558909 | 0.4114895 | 0.33554177 | 0.4919014 |
| **duration of diabetes** | 1.064972 | 1.007983 | 1.528812 | 1.031975 | 0.9389918 | 0.65410285 | 0.9920805 |
| **VFA** | 6.769868 | 5.439918 | 8.498195 | 2.601897 | 0.1477134 | 0.11767205 | 0.1838263 |
| **Average daily intake of rice** | 1.246077 | 1.118463 | 1.511162 | 1.116278 | 0.8025189 | 0.66174231 | 0.8940843 |
| **Hemoglobin** | 1.028442 | 1.000322 | 3.509435 | 1.014121 | 0.9723450 | 0.28494613 | 0.9996778 |
| **Urea** | 1.155685 | 1.056393 | 1.429805 | 1.075028 | 0.8652877 | 0.69939588 | 0.9466178 |
| **Creatinine** | 1.288104 | 1.148970 | 1.557185 | 1.134947 | 0.7763349 | 0.64218452 | 0.8703448 |
